# Colquhounia Root Tablet Modulates Psoriatic Immune Responses Involving NF-κB-Driven Dendritic-Cell Maturation and Th17/Treg Imbalance: An Integrative Network Pharmacology and Transcriptomic Study

**DOI:** 10.3390/pharmaceutics18091105

**Published:** 2026-09-02

**Authors:** Qingqing Xu, Lisong Sheng, Hui Zhao, Lingyun Du, Jingjing Wei, Huijie Zhang, Tianyu Zhang, Huanhuan Zhang, Chunhong Zhang, Rong Sun

**Affiliations:** 1 Department of Dermato-Venereology, The Second Qilu Hospital of Shandong University, Jinan 250033, China; 201662017218@email.sdu.edu.cn (Q.X.); weijingjing1999@163.com (J.W.); handsome2688@126.com (H.Z.); 2 Basic Medical Research Institute, Integrated Traditional Chinese and Western Medicine Center, The Second Qilu Hospital of Shandong University, Jinan 250033, China; huijiezhang6@163.com (H.Z.); zty17860505903@163.com (T.Z.); 3 Advanced Medical Research Institute, Cheeloo College of Medicine, Shandong University, Jinan 250012, China; lisanoid1@gmail.com (L.S.); 202294000076@sdu.edu.cn (H.Z.); dulingyunjiayou@163.com (L.D.); 4 National Administration of Traditional Chinese Medicine, High-Level Key Disciplines of Traditional Chinese Medicine, Toxicology of Traditional Chinese Medicine, Cheeloo College of Medicine, Shandong University, Jinan 250012, China

**Keywords:** Colquhounia Root Tablet, psoriasis, NF-κB signaling, dendritic-cell maturation, Th17/Treg imbalance, network pharmacology

## Abstract

**Background/Objectives**: Psoriasis is a chronic inflammatory skin disease driven by Th17/Treg imbalance. Colquhounia Root Tablet (CRT), derived from *Tripterygium hypoglaucum*, has shown clinical potential for psoriasis, but its mechanisms remain unclear. This study aimed to evaluate the anti-psoriatic effects of CRT and elucidate its underlying mechanisms. **Methods**: Anti-psoriatic activity was evaluated in an IMQ-induced psoriasis-like mouse model. Mice received oral CRT at 0.085, 0.17, or 0.35 g/kg daily from days 2 to 8. Immune-cell populations were analyzed by flow cytometry. Bone marrow-derived dendritic cells (BMDCs) were used for in vitro studies. Network pharmacology, transcriptomics, molecular docking, and experimental validation were integrated to explore the mechanisms. **Results**: CRT dose-dependently ameliorated psoriasiform dermatitis and reduced Th17/Treg ratio while inhibiting CD11c^+^MHC II^+^ DC activation in vivo. In vitro, CRT suppressed R848-induced BMDC maturation and inhibited p65/IκBα phosphorylation. Transcriptomic analysis revealed modulation of TNF, NF-κB, IL-17, and JAK-STAT pathways. Molecular docking predicted the strong binding of multiple CRT compounds to RELA. **Conclusions**: CRT exerts anti-psoriatic effects in a murine model with concurrent modulation of NF-κB-related DC maturation and Th17/Treg correction, suggesting a potential immunomodulatory mechanism requiring further causal validation.

## 1. Introduction

Psoriasis is a chronic immune-mediated inflammatory skin disease characterized by silvery white scales, erythematous plaques, and pruritus. Beyond cutaneous manifestations, it is associated with systemic comorbidities, including inflammatory arthritis, cardiovascular diseases, and metabolic disorders [1]. The disease arises from dysregulated immune responses triggered by genetic predisposition and environmental factors (e.g., infections), leading to aberrant keratinocyte proliferation, angiogenesis, and infiltration of immune cells (including DCs, T cells, and neutrophils). A central mechanism driving psoriatic inflammation is the disruption of Th17/Treg immune homeostasis, characterized by an increased Th17/Treg ratio and functional impairment of regulatory T cells (Tregs) in both peripheral blood and lesional skin [2]. Current therapies (immunosuppressants, biologics, phototherapy) target inflammation and epidermal hyperplasia but face limitations like incomplete efficacy, drug dependence, and long-term safety concerns. Thus, exploring alternative treatments that effectively restore immune homeostasis remains clinically imperative.

Ethnopharmacology, as a vital component of China’s traditional medical system, demonstrates unique advantages in the treatment of chronic inflammatory diseases. Colquhounia Root Tablet (CRT), also named HuoBaHuaGenPian or Kunming Shanhaitang Pian, is a Chinese traditional medicine made from the peeled root of Tripterygium hypoglaucum (Lévl.) Hutch (THH). THH was first documented in the “Diannanbencao” (China’s earliest local herbal monograph, depicting over 400 types of herbs from the southwestern plateau region) written by Lan Mao during the Ming Dynasty. According to Traditional Chinese Medicine theory, THH exhibits functions such as dispelling wind, removing dampness, and promoting blood circulation. In addition, experimental studies have demonstrated its anti-inflammatory and analgesic effects [3]. Owing to its high efficacy and low toxicity, preliminary clinical studies have suggested that CRT may be beneficial in the treatment of autoimmune diseases, kidney diseases [4], and skin disorders [5,6], although further high-quality clinical trials are required to confirm these findings. It contains several terpenoids, berberine, alkaloids, steroids, and lactones, such as triptolide, triptophenolide and epicatechin [7]. Pharmacokinetic studies have shown that CRT exerts immunosuppressive, anti-inflammatory, and analgesic effects, with low toxicity in the treatment of rheumatic diseases [3]. To date, CRT has been approved for rheumatoid arthritis and systemic lupus erythematosus [8,9] and has also shown significant clinical potential for psoriasis and palmoplantar pustulosis [5,10].

Recently, Fei Li et al. [11] reported that topical application of Colquhounia Root alleviates skin inflammation and pruritus in IMQ-induced psoriasiform dermatitis by suppressing M5-induced inflammatory cytokines and itch-related molecules in keratinocytes. However, several critical gaps remain: ① the administration route used was topical, whereas CRT is clinically administered **orally** as a tablet; ② the study did not elucidate the underlying signaling pathways, particularly regarding systemic immune regulation. Consequently, the mechanisms of action of CRT via its primary clinical route (oral administration) remain largely unexplored, warranting systematic investigation. In the present study, we integrated network pharmacology, transcriptomic analysis and in vivo and in vitro experimental validation to elucidate the molecular mechanisms underlying CRT’s anti-psoriatic activity.

## 2. Material and Methods

### 2.1. Animals

We purchased 6–8 weeks male Balb/c mice from Beijing Huafukang Biotechnology Co., Ltd. (Beijing, China). All mice were housed in a pathogen-free facility under controlled conditions (temperature: 25 ± 5 °C; humidity: 55 ± 5%) with free access to a standard laboratory diet and water. All animal experiments were conducted in accordance with the Guide for the Care and Use of Laboratory Animals and were approved by the Animal Care and Use Committee of the Second Qilu Hospital of Shandong University (KYLL- KYLL2024630, approved on 4 June 2024).

### 2.2. Reagents and Treatments

CRT was obtained from Sinomune Pharmaceutical Co., Ltd. (Chongqing, China). For chemical characterization, CRT was subjected to UPLC-Q-TOF-MS analysis. The established fingerprint revealed 18 major components, including gallic acid, procyanidin B, epigallocatechin, and triptoquinone A, with batch-to-batch similarities ranging from 0.992 to 0.999 [12]. CRT tablets were ground into powder and dissolved in double-distilled water at 0.1 g/mL to obtain a stock solution of 100 mg/mL. IMQ cream was purchased from Sichuan Med-Shine Pharmaceutical Co., Ltd. (5%; Mingxinlidi, Chengdu, China). Methotrexate (MTX) tablets were purchased from Sine Pharmaceutical Laboratories Co., Ltd. (Shanghai, China; catalog No. H31020644). Mice were given 0.2 mL of solution by oral administration per 20 g of body weight.

A total of 36 male BALB/c mice (6–8 weeks old) were randomly divided into six groups (N = 6 per group). An additional 12 mice were used in preliminary dose-finding experiments to determine the optimal doses. Except for the control group, all mice received a daily topical dose of 62.5 mg 5% IMQ cream after having the dorsal skin on the dorsum shaved for 8 consecutive days to induce psoriasis-like lesions. From day 2 to day 8, mice were treated once daily with either vehicle (IMQ group), methotrexate (MTX; 0.5 mg/kg), or CRT tablets at low (0.085 g/kg), medium (0.17 g/kg), or high (0.35 g/kg) doses. These doses were selected based on the clinical dose recommended for rheumatoid arthritis (1.62–2.70 g/day for a 70 kg adult), converted into mouse equivalent doses using body surface area normalization [8]. On day 9, blood was collected for lymphocyte analysis, and skin and spleen samples were harvested after euthanasia.

### 2.3. Scoring Severity of Skin Inflammation

The signs of psoriasis-like skin lesions were evaluated daily using a modified PASI-like scoring system adapted for the murine model. Erythema and scaling were scored independently on a scale from 0 to 4 as follows: 0, none; 1, slight; 2, moderate; 3, marked; and 4, very marked. The total score (erythema plus scaling, range 0–8) was calculated as a measure of inflammation severity. The scoring was performed by two independent observers but was not conducted in a blinded manner.

### 2.4. Skin and Spleen Histology and Immunochemical Staining

The back-skin samples were fixed in paraformaldehyde, subsequently dehydrated and embedded in paraffin. Skin sections (5 μm) were stained with hematoxylin and eosin (H&E) and Ki67 antibodies (ab279653, Abcam, Cambridge, UK). Staining was scanned by a NanoZoomer Digital Pathology scanner (Hamamatsu Photonics K.K., Hamamatsu City, Japan). For epidermal thickness quantification, three non-adjacent sections per animal were selected, and five randomly chosen fields per section were analyzed. Epidermal thickness was measured at three equidistant points per field using NDPView 2 software, and the average value was calculated for each mouse. Ki67-positive cells were quantified by counting the number of positive cells per millimeter of basement membrane in five randomly selected fields per section. The quantification was performed by two independent observers in a blinded manner.

### 2.5. Flow Cytometry Analysis of Immune-Cell Populations

Peripheral blood lymphocytes. For B- and T-cell subset analysis, 100 µL of anticoagulant blood from each mouse was stained with the following surface antibodies for 30 min at room temperature in the dark: APC anti-CD3 (clone 17A2, cat. No. E-AB-F1013E; Elabscience, Paris, France), FITC anti-CD19 (clone 6D5, cat. No. E-AB-F0986C; Elabscience), PE anti-CD45R/B220 (clone RA3-6B2, cat. No. E-AB-F1112D; Elabscience), FITC anti-CD4 (clone GK1.5, cat. No. E-AB-F1097C; Elabscience), and PE anti-CD8a (clone 53-6.7, cat. No. E-AB-F1104D; Elabscience), each used in 3–5 µL volumes per sample. After staining, erythrocytes were lysed using BD FACS Lysing Solution (BD Biosciences, San Jose, CA, USA) for 5 min at room temperature. Cells were washed twice with PBS and analyzed with flow cytometry (Beckman Coulter, Brea, CA, USA). Lymphocytes were gated based on FSC-A/SSC-A, followed by CD3^+^ T cells, from which CD4^+^ and CD8^+^ subpopulations were analyzed; B cells were identified as CD19^+^ or B220^+^ cells within the lymphocyte gate.

Splenic Treg, Th17, and dendritic cells. Spleens were gently ground on a 200-mesh stainless steel sieve to prepare single-cell suspensions. Pooled cells were treated with red blood cell lysis buffer (BD FACS Lysing Solution), washed with RPMI-1640 medium, and counted. For the surface staining of Treg and Th17 cells, cells were incubated with FITC anti-CD4 (clone GK1.5, cat. No. E-AB-F1097C; Elabscience, 1:200) and APC anti-CD25 (clone PC61, cat. No. 17-0251-82; eBioscience, San Diego, CA, USA; 1:100) for 30 min at 4 °C in the dark. For dendritic-cell surface staining, cells were incubated with PE anti-CD11c (clone N418, cat. No. E-AB-F0991D; Elabscience), FITC anti-MHC II (clone M5/114.15.2, cat. No. E-AB-F0990C; Elabscience), and PE-Cy7 anti-CD86 (clone GL-1, cat. No. E-AB-F0992H; Elabscience), each used in 3–5 µL volumes per sample. For Treg detection, cells were fixed and permeabilized using the Foxp3/Transcription Factor Staining Buffer Set (eBioscience) according to the manufacturer’s instructions, followed by intracellular staining with PE anti-Foxp3 (clone FJK-16s, cat. No. 12-5773-82; eBioscience; 1:100). For Th17 detection, cells were fixed, permeabilized, and stained intracellularly with PE anti-IL-17A (clone eBio17B7, cat. No. 12-7177-81; eBioscience; 1:100) without prior PMA/ionomycin stimulation. Stained cells were acquired on a flow cytometer (Beckman Coulter, Brea, CA, USA) and analyzed using FlowJo_V10 software (BD Biosciences). The gating strategy for Tregs was: lymphocytes → CD4^+^ → CD25^+^Foxp3^+^; for Th17 cells: lymphocytes → CD4^+^ → IL-17A^+^; for dendritic cells: lymphocytes → CD11c^+^ → MHC II^+^CD86^+^.

### 2.6. Network Pharmacology Analysis

The chemical constituents of CRT were obtained from a previously published UPLC-Q-TOF-MS study [7] and their SMILES or formulas from PubChem. Ingredient targets were obtained from TCMSP, STITCH (highest confidence score: 0.900), and SwissTargetPrediction (gene probability >0.6, Homo sapiens). Psoriasis-related target genes were retrieved from Genecards and DisGeNET using “Psoriasis” (with a relevance score >6). Overlapping genes between compound and psoriasis targets were identified via the Venn platform. A compound–target interaction network was constructed and analyzed using Cytoscape 3.9.0 software. GO and KEGG pathway enrichment of overlapping genes was performed using the metascape platform, with bubble charts plotted using the online platform at http://www.bioinformatics.com.cn/, accessed on 23 August 2026.

### 2.7. Real-Time Polymerase Chain Reaction (RT-PCR)

Total RNA was extracted from cells using the SteadyPure Quick RNA Extraction kit (AG21023, Accurate Bio, Changsha, China) according to the manufacturer’s instructions. cDNA was synthesized from the RNA using the HiScript QRT SuperMix for qPCR Kit (R223-01, Vazyme, Nanjing, China). Quantitative PCR (qPCR) analysis was performed on a real-time PCR system (Eppendorf, Hamburg, Germany) using the SYBR Green premix Pro Taq HS qPCR kit (AG11701, Accurate Bio). Target gene expression levels were normalized to the level of β-actin, and relative changes were calculated using the ΔΔCt method. The primer sequences are listed in Table 1.

### 2.8. Effect of CRT on Induction and Culture of BMDCs

BMDCs were isolated by flushing the femurs and tibiae of 6- to 8-week-old BALB/c mice with PBS (one donor per independent experiment; n = 3 experiments). Pooled cells from the legs were treated with red blood cell lysis buffer (Solarbio, Beijing, China) and washed with RPMI-1640 medium, and cultured in 96-well plates (1 × 10^4^/well) or 6-well plates (1 × 10^6^/well) with RPMI-1640 medium supplemented with 10% fetal bovine serum (FBS) (Gibco, Waltham, MA, USA), 1% penicillin–streptomycin, 20 ng/mL IL-4 and 20 ng/mL GM-CSF (PeproTech, Rocky Hill, NJ, USA). The medium was replaced every other day, and cells were cultured for 7 days to induce differentiation into BMDCs.

On day 8, BMDC viability was assessed using the CCK-8 assay (Solarbio, Beijing, China). Cells were seeded in 96-well plates and treated with various concentrations of CRT (0–200 µg/mL) for 24 h, followed by CCK-8 incubation for 2 h. Absorbance was measured at 450 nm using a microplate reader (BioTek, Winooski, VT, USA).

For maturation experiments, BMDCs were seeded in 6-well plates and pre-treated with CRT for 2 h, followed by stimulation with 1 µg/mL R848 (TLR7/8 agonist) for 24 h to induce DC maturation. After treatment, cells were harvested, washed with PBS, and stained with anti-CD11c-PE, anti-MHC II-FITC, anti-CD80-PE-Cy7, and anti-CD86-APC antibodies at 4 °C for 30 min in the dark. These antibodies are the same as those described in Section 2.5 for splenic DC staining. Cells were then washed twice with PBS and analyzed by flow cytometry. The CD11c-positive population was gated, and the expression levels of MHC II, CD80, and CD86 were evaluated within this gate.

### 2.9. Western Blot Assay

After treatment with chemicals, cells were lysed in RIPA lysis buffer (Beyotime, Shanghai, China; P0013B) supplemented with protease/phosphatase inhibitor. After they were sonicated and centrifuged at 12,000× *g* for 15 min at 4 °C to remove insoluble debris, protein concentrations were determined using a BCA protein assay kit (Beyotime, P0012), and 20 μg of protein per lane was resolved by sodium dodecyl sulfate–polyacrylamide gel electrophoresis (SDS-PAGE) on 8% or 10% separating gels with 5% stacking gels. The separated proteins were then electrophoretically transferred onto 0.2µM Nitrocellulose membranes (Cytiva, Marlborough, MA, USA; 10600001) using a wet transfer system at 250 mA for 2 h at 4 °C. After being blocked with 5% (*w*/*v*) non-fat dry milk in TBST buffer, the blots were incubated with primary antibodies against IκB alpha (Affinity, San Francisco, CA, USA; AF5002; 1:1000), p-IKBa (Ser32) (CST; 2859; 1:1000), NF-κB p65 (CST; 8242; 1:2000), phosphor-NF-κB p65 (Ser536) (CST; 3033; 1:2000), phosphor-Stat3 (Tyr705) (CST; 9145; 1:1000), Stat3 (Santa Cruz Biotechnology, Dallas, TX, USA; sc-8019; 1:500), Erk1/2 (CST; 4695,), phosphor-Erk1/2 (Thr202/Tyr204) (CST; 4370; 1:1000) and β-actin (Proteintech; sc-8432; 1:3000) overnight at 4 °C, followed by incubation with appropriate peroxidase-conjugated secondary antibodies (SeraCare, Milford, MA, USA; 5220-0336 or 5220-0341; 1:5000). β-Actin was used as an internal control. The detection system visualization (Millipore, Burlington, MA, USA) was followed by exposure to a Chemiluminescence Imaging Analyzer (Tanon 5200, Shanghai, China). The protein bands were then analyzed using ImageJ software. The grayscale values of the detected proteins were normalized to the values of the corresponding actin to determine changes in protein expression. All Western blot experiments were performed with three independent biological replicates (n = 3). The phosphorylation levels of each target protein were normalized to their corresponding total protein levels, and the ratios of phosphorylated to total protein (p-Stat3/Stat3, p-IκBα/IκBα, p-p65/p65, and p-Erk1/2/Erk1/2) were calculated and statistically analyzed. Data are presented as means ± SEM (n = 3).

### 2.10. Immunofluorescence Staining

Cell climbing sheets with different treatment were fixed with ice-cold methanol/acetone (1:1) and incubated with 3% bovine serum albumin in PBS with 0.1% Triton X-100 for 25 min. After washing in PBS with 0.01% Triton X-100, immunofluorescence staining was performed using primary antibodies against p-NF-κB p65 (CST; 3033; 1:500) and p-Erk1/2 (CST; 4370; 1:200) and then probed with secondary antibodies (Abcam; ab150077; 1:1000). Fluorescent staining images were acquired by a PerkinElmer Mantra multispectral imaging microscope (PerkinElmer, Shelton, CT, USA) or by laser confocal microscopy (LSM780, Zeiss, Oberkochen, Germany). For quantification, five random fields per group were captured under identical settings. Mean fluorescence intensity (MFI) of p-p65 and p-Erk1/2 was measured using ImageJ and normalized to the number of DAPI-positive nuclei. Data are expressed as relative fluorescence intensity normalized to the control group.

### 2.11. Molecular Docking

To explore the binding modes between RelA and CRT compounds, molecular docking was performed using AutoDock Vina (v1.2.0). The RelA protein structure (PDB ID: 3QXY) was retrieved from the RCSB Protein Data Bank, and ligand structures were obtained from PubChem without further optimization. Docking areas were defined based on the protein’s binding pockets, and the lowest binding energy conformation was selected. A binding energy below −7 kcal/mol was considered to indicate a favorable predicted interaction [13]. Visualization of docking poses was conducted using PyMOL and Discovery Studio.

### 2.12. Statistical Analysis

Data are expressed as means ± SEM for all treatment comparisons. Baseline characteristics (e.g., body weight) are presented as means ± SD. Throughout the manuscript, N denotes the number of animals per group (biological replicates), while n denotes the number of independent experimental replicates (technical replicates). Sample sizes were determined by power analysis using G*Power software (version 3.1) based on preliminary data (effect size = 1.8, SD = 0.5; two-sided α = 0.05; power = 0.8), yielding a minimum of 5 mice per group; a total of 6 mice were included to account for potential attrition. For in vitro experiments, all assays were performed with at least three independent replicates (n = 3).

Before statistical analysis, normality was assessed using the Shapiro–Wilk test, and homogeneity of variances was examined using Levene’s test. For comparison among more than two groups, one-way analysis of variance (ANOVA) was performed only when data met both assumptions (*p* > 0.05), followed by Dunnett’s post hoc test for multiple comparisons against the IMQ-induced model group or the untreated control group, as indicated in each figure legend. For ordinal data (e.g., PASI scores) or data that violated normality assumptions, the Kruskal–Wallis H test was used, followed by Dunn’s post hoc test with the IMQ group or the control group as the reference, as appropriate. Comparisons between two groups were performed with Student’s unpaired *t*-test where applicable. All statistical analyses were conducted using GraphPad Prism software (San Diego, CA, USA). Statistical significance was set at *p* < 0.05, and *p* < 0.01 or *p* < 0.001 was considered highly significant. All significance symbols (*, #, etc.) are defined in the corresponding figure legends.

## 3. Results

### 3.1. Identification of CRT-Derived Bioactive Compounds and Potential Target Prediction

**Figure 1:**
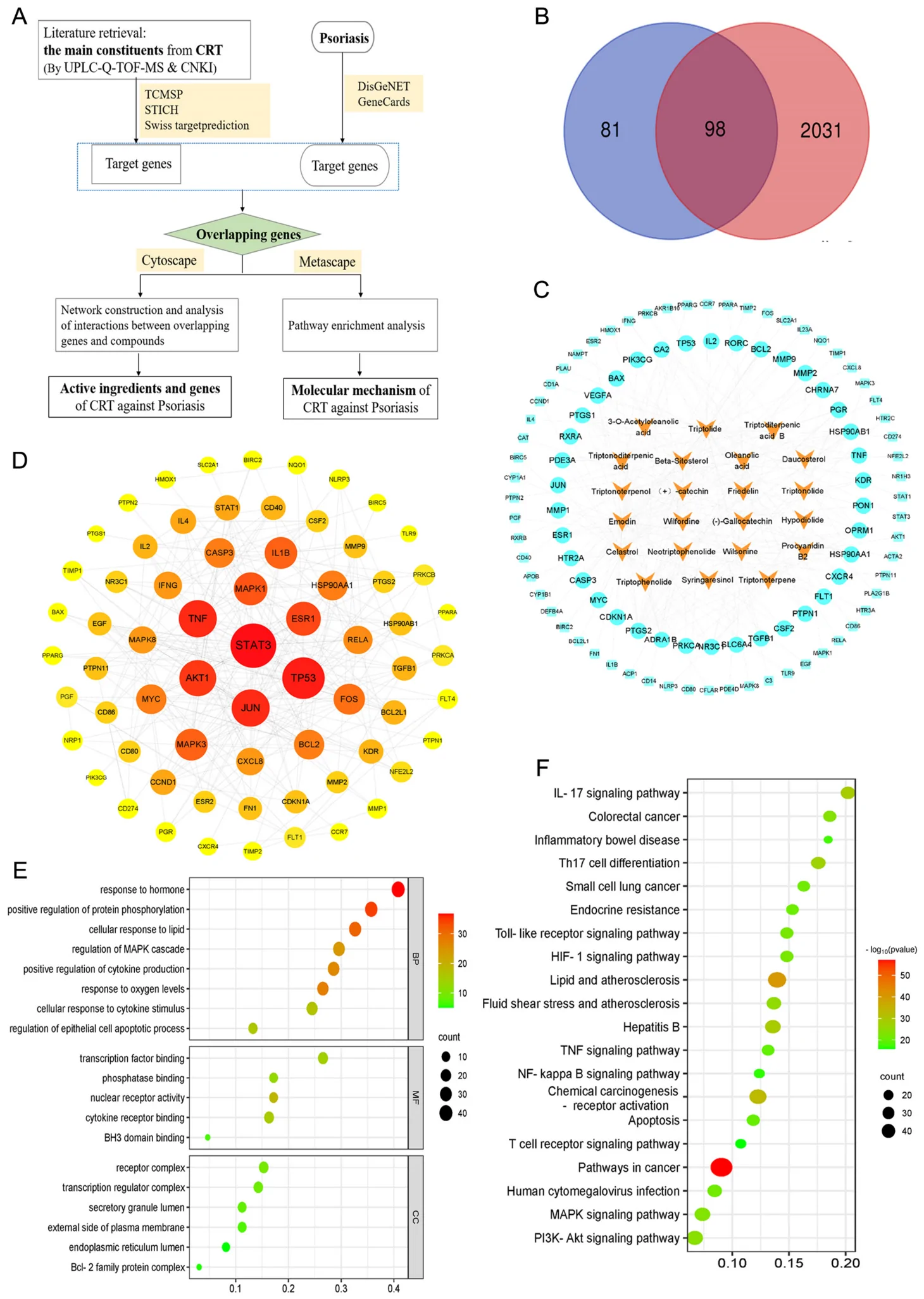
Bioactive constituents and predicted targets of CRT. (**A**) Schematic of the analytical workflow. (**B**) Venn diagram illustrating targets shared between CRT and psoriasis. (**C**) Network of compound–target interactions for the anti-psoriasis effect of CRT. (**D**) Protein–protein interaction (PPI) network of common targets. The color of a node represents the degree value. (**E**) Gene Ontology (GO) enrichment analysis of common targets. The *X*-axis shows the ratio of enriched to background genes; the *Y*-axis lists the biological processes. (**F**) Top 20 enriched KEGG pathways presented as a dot plot. The *Y*-axis lists the pathway terms.

To elucidate the pharmacological basis of CRT’s anti-psoriasis activity, we performed an integrated network pharmacology analysis. The overall workflow is depicted in Figure 1A. A total of 43 compounds previously reported in CRT [7], along with the main ingredients identified by UPLC-Q Exactive MS analysis (Supplementary Figure S1), were included for further investigation. Using multiple database platforms (TCMSP, STITCH, and SwissTargetPrediction), we predicted target genes associated with these compounds. The analysis revealed that 23 bioactive constituents were linked to 179 potential gene targets (Supplementary Table S1).

A comprehensive search of the DisGeNET and GeneCards databases yielded 2129 potential psoriasis-related targets. Intersection analysis using a Venn diagram revealed 98 overlapping target proteins shared between CRT and psoriasis (Figure 1B and Supplementary Table S2), suggesting their potential role in mediating the anti-psoriasis effects. Using Cytoscape 3.9.0, we generated a compound–target interaction network comprising 120 nodes and 216 edges (Figure 1C), highlighting key compounds with multi-target activity and targets influenced by multiple compounds. Furthermore, protein–protein interaction (PPI) network analysis identified key targets, including STAT3, TP53, TNF, AKT1, JUN, MAPK3, and RELA (Figure 1D). GO enrichment revealed CRT involvement in hormone- and lipid-related responses, MAPK cascade regulation, protein phosphorylation, and cytokine production (Figure 1E). KEGG pathway analysis further indicated that CRT modulates the IL-17, Toll-like receptor, TNF, NF-κB, and Th17 differentiation pathways (Figure 1F). These findings collectively suggest that CRT exerts its anti-psoriatic effects largely through modulation of inflammatory responses, particularly Th17 cell differentiation.

### 3.2. CRT Ameliorated IMQ-Induced Psoriasis-like Inflammation in Dose-Dependent Manner

**Figure 2:**
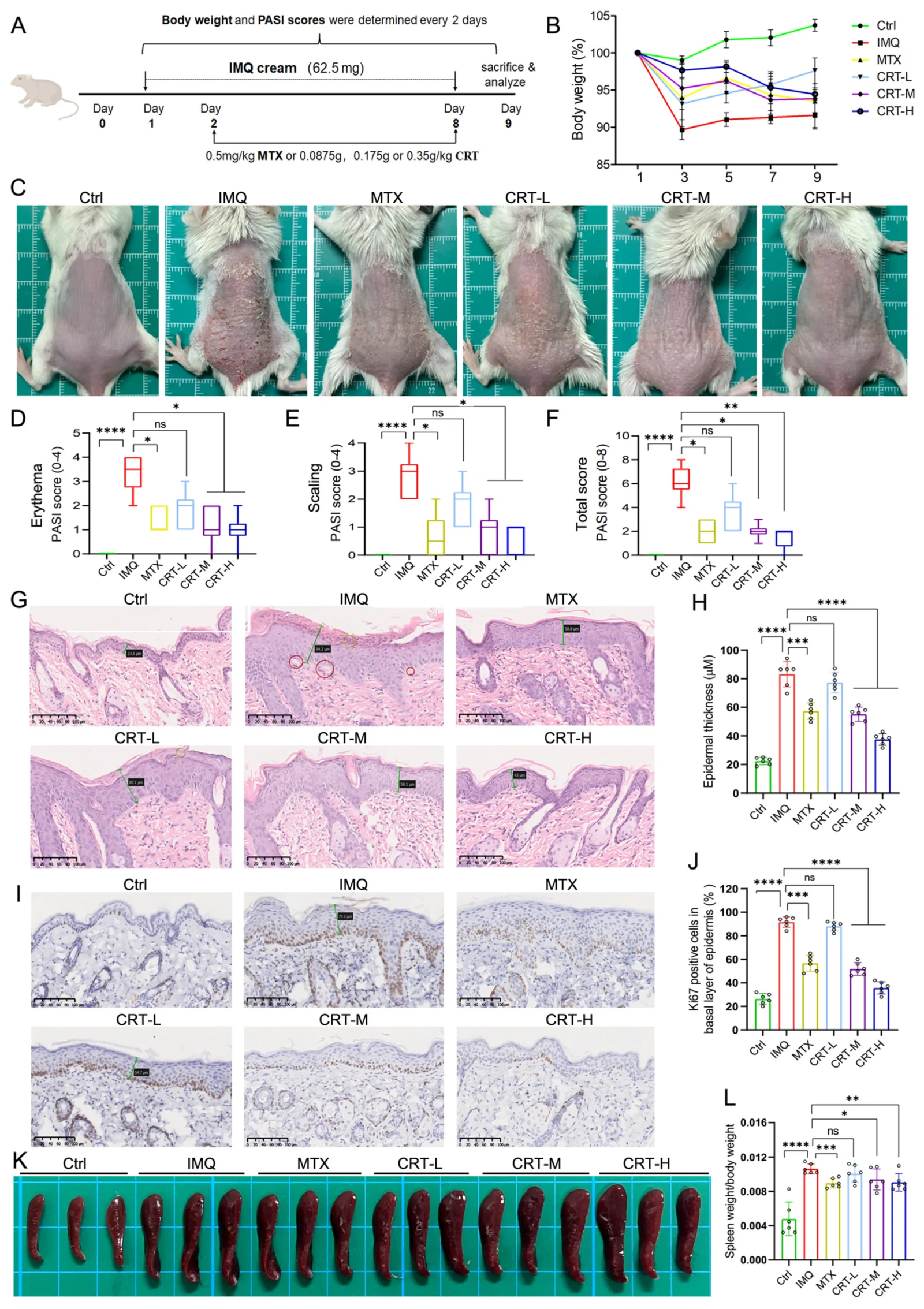
CRT treatment attenuated IMQ-induced psoriasis-like lesions in Balb/c mice. (**A**) Experimental schedule (detailed in Section 2). (**B**) Change in body weight during the medication process. (**C**) Dorsal skin lesions from each experimental group. (**D**–**F**) Psoriasis Area and Severity Index (PASI) scores for erythema, scaling, and total on day 9. Data are shown as medians with interquartile ranges (IQRs), and statistical significance was determined by the Kruskal–Wallis H test followed by Dunn’s post hoc test. (**G**) Representative H&E-stained sections (×200). (**H**) Quantification of epidermal thickness. (**I**) Immunohistochemical staining for Ki67 (×200; scale bar = 50 μm). (**J**) Percentage of Ki67-positive basal epidermal cells. (**K**) Spleen images. (**L**) Spleen index (spleen weight/body weight). For panels (**H**,**J**,**L**), data are means ± SEM (N = 6 mice per group); statistical comparisons were performed using one-way ANOVA followed by Dunnett’s post hoc test against the IMQ group. * *p* < 0.05, ** *p* < 0.01, *** *p* < 0.001 and **** *p* < 0.0001 vs. indicated group.

On day 3, all groups showed weight loss; the IMQ group had the most significant decrease (~10%), while the control and CRT-H groups showed milder reductions (Figure 2B). Thereafter, weights in the IMQ and CRT-L groups gradually increased, whereas the MTX, CRT-M and CRT-H groups continued decreasing but remained above IMQ levels. Erythema and scaling appeared on the dorsal skin by day 3 and worsened over time. MTX and CRT treatments delayed and reduced lesion development. By day 9, the IMQ group developed severe erythema and silvery scales; MTX- and CRT-L-treated mice showed mild erythema and minimal scaling, while the CRT-M and CRT-H groups exhibited no visible lesions (Figure 2C–F). Psoriasis-like severity decreased with CRT in a dose-dependent manner.

HE staining (Figure 2G) revealed that IMQ induced hyperkeratosis, parakeratosis, epidermal rete ridge extension, inflammatory infiltration, and capillary congestion. Oral MTX and CRT-M/H markedly ameliorated these changes, with normalized keratinization, reduced epidermal thickness (IMQ: 82.5 ± 9.8 μm; MTX: 57.6 ± 6.5 μm; CRT-L: 77.1 ± 8.1 μm; CRT-M: 54.9 ± 5.5 μm; CRT-H: 37.5 ± 4.5 μm; Figure 2H), and reduced dermal inflammation (Figure 2G). The spleen index (Figure 2K,L) was also attenuated, especially in the MTX and CRT-M/H groups. Overall, CRT at medium/high doses exerted therapeutic effects comparable or even superior to MTX, alleviating both clinical and histopathological features of IMQ-induced psoriasis-like dermatitis in a dose-dependent manner.

### 3.3. CRT Corrects IMQ-Induced Th17/Treg Imbalance and Modulates Immune Profiles

A central mechanism driving psoriatic inflammation is the disruption of Th17/Treg immune homeostasis, characterized by an increased Th17/Treg ratio and functional impairment of Tregs [14]. To assess the immunomodulatory effects of CRT, we analyzed immune-cell populations in the spleen and peripheral blood.

**Figure 3:**
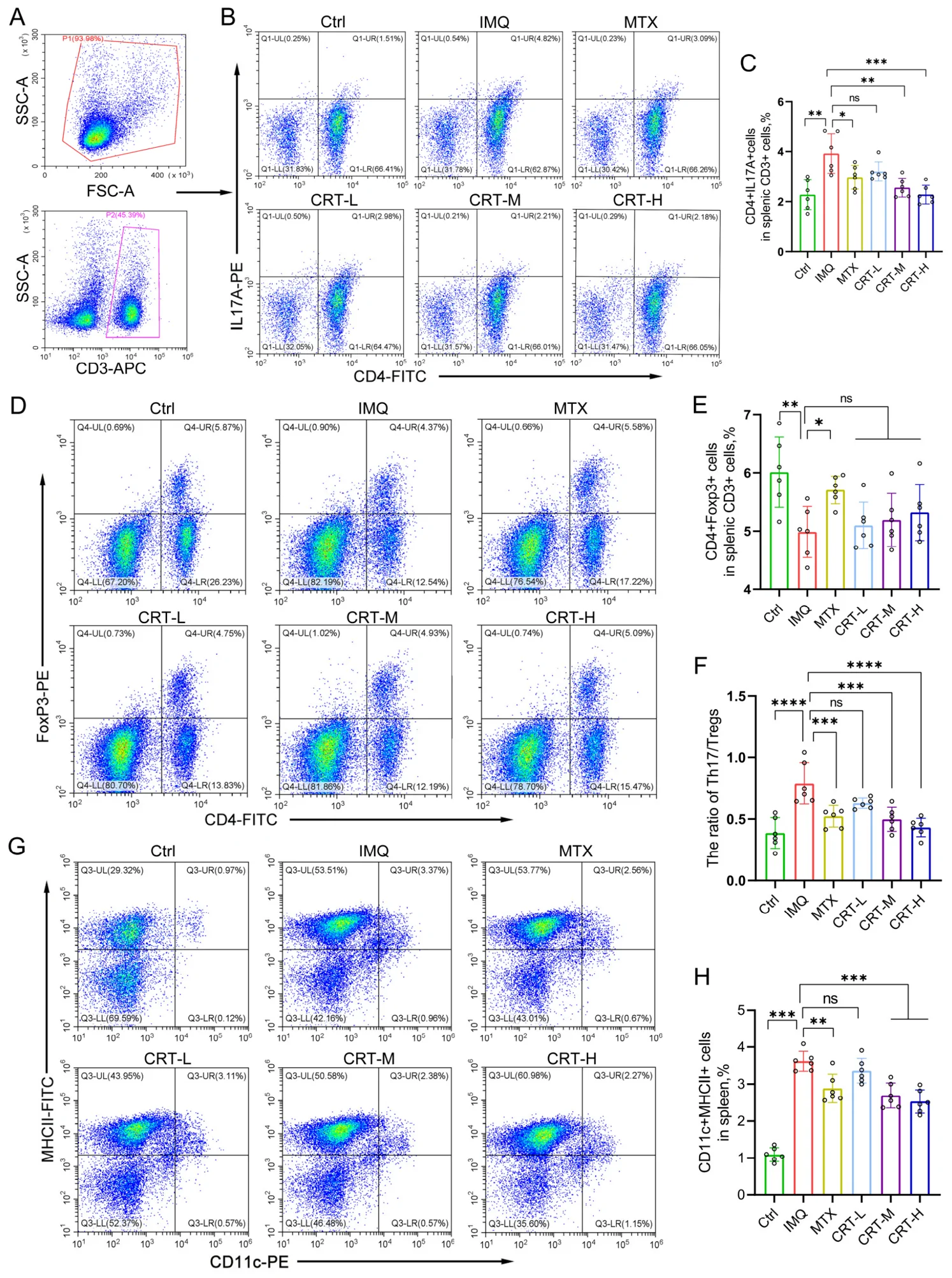
CRT corrects IMQ-induced Th17/Treg imbalance and dendritic-cell differentiation. (**A**) Gating scheme for identifying CD3^+^ T cells in mouse spleen. (**B**) Representative flow cytometry images showing CD4^+^IL-17A^+^ T cells within splenic CD3^+^ T populations across experimental groups. (**C**) Statistical analysis of the proportion of CD4^+^IL-17A^+^ T cells among CD3^+^ T cells. (**D**) Flow cytometry analysis of CD4^+^Foxp3^+^ Treg cells in mouse spleen. (**E**) Quantitative assessment of CD4^+^Foxp3^+^ Treg cell frequency. (**F**) The rate of Th17 cells to Treg cells in spleen was analyzed statistically. (**G**) Representative flow cytometric images for CD11c+MHCII+ DCs in mouse spleen from different groups. (**H**) The rate of CD11c+MHC II+ DC expression were analyzed statistically. Data are shown as means ± SEM (N = 6). Statistical significance is denoted by * *p* < 0.05, ** *p* < 0.01, *** *p* < 0.001, and **** *p* < 0.0001 vs. indicated group, respectively.

Splenic CD3^+^ T cells were isolated (Figure 3A) and the proportions of Th17 and Treg cells examined by flow cytometry. As shown in Figure 3B,C, flow cytometry revealed a significant increase in the percentage of CD4^+^IL-17^+^ Th17 cells in IMQ-treated mice compared with controls. MTX treatment slightly reduced this proportion, while CRT dose-dependently decreased the Th17 population. Although IMQ exposure reduced the frequency of CD4^+^Foxp3^+^ Treg cells—an effect partially restored by MTX—CRT did not significantly alter Treg percentages (Figure 3D,E). However, CRT at medium and high doses substantially lowered the Th17/Treg ratio relative to the IMQ group (Figure 3F). Given the role of DCs in Th17 differentiation, we also examined splenic DCs. The IMQ group exhibited a marked elevation in CD11c^+^MHC II^+^ DCs, which was more effectively suppressed by CRT-M/H treatment than by MTX (Figure 3G,H).

**Figure 4:**
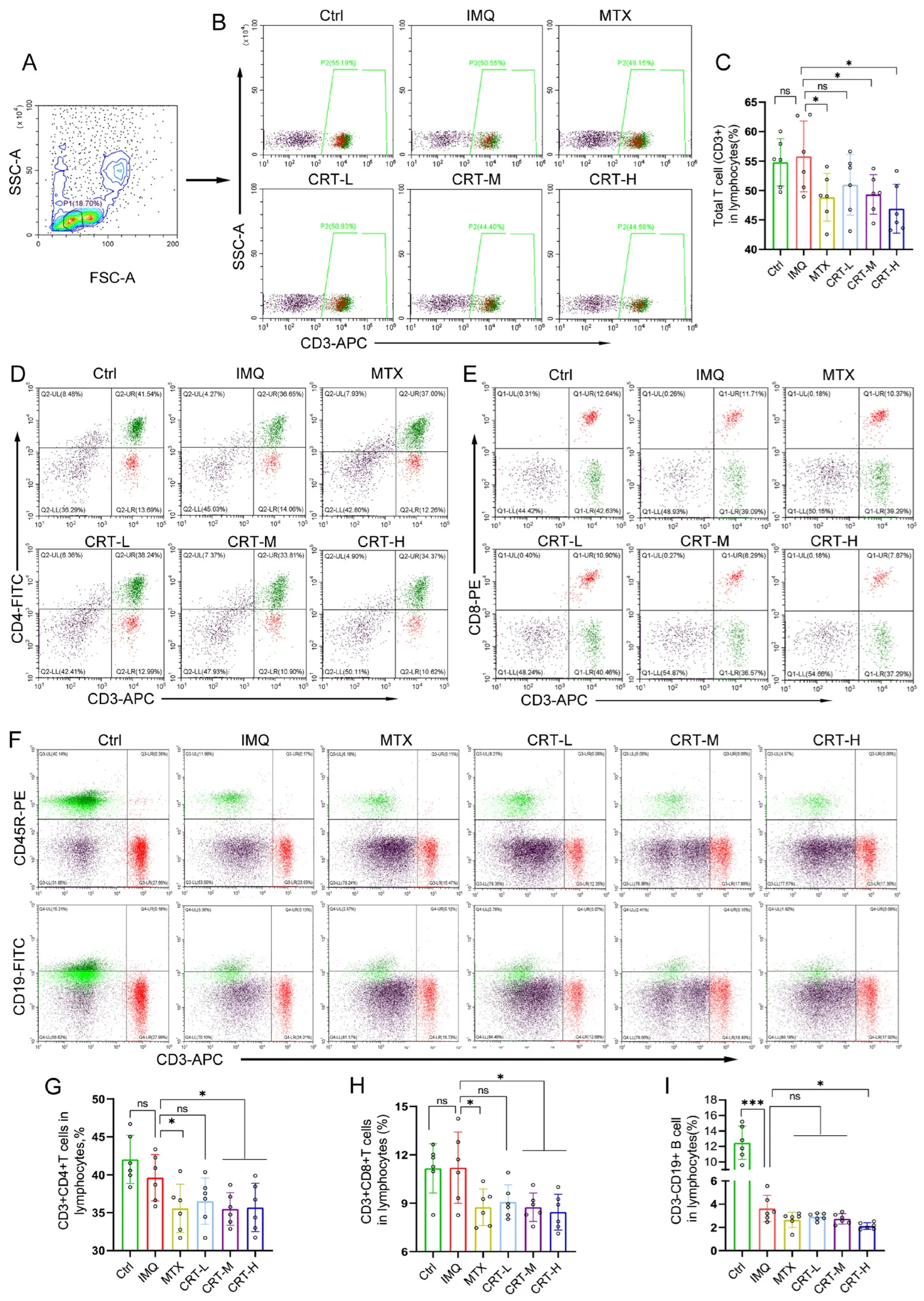
CRT treatment adjusted IMQ-induced immune response in peripheral blood. (**A**) Gating scheme for identifying lymphocyte populations in peripheral blood. (**B**) Representative flow cytometry images for CD3^+^T cells plots depicting CD3^+^ T cells. (**C**) Statistical analysis of the proportion of total T cells across groups. (**D**,**E**) Flow cytometric profiles showing the expression of (**D**) CD3^+^CD4^+^ and (**E**) CD3^+^CD8^+^ T-cell subsets. (**F**) Representative dot plots illustrating the staining for CD3^−^CD45R^+^ and CD3^−^CD19^+^ B cells. (**G**–**I**) Quantification of the percentages of (**G**) CD3^+^CD4^+^ T cells, (**H**) CD3^+^CD8^+^ T cells and (**I**) CD3-CD19^+^ B cells in lymphocytes. Data are presented as means ± SEM (N = 6). Significance levels: * *p* < 0.05, and *** *p* < 0.001 vs. indicated group.

We also evaluated the effect of CRT on peripheral immune-cell subsets. While IMQ treatment did not markedly alter overall lymphocyte proportions, MTX and CRT-M/H modestly reduced total lymphocyte counts (Figure 4A–C). Changes in CD4^+^ and CD8^+^ T-cell subsets mirrored those of total CD3^+^ T cells across groups (Figure 4D,E,G,H). CRT-M/H showed lymphocytosis-inhibitory effects comparable to MTX. Notably, IMQ significantly decreased the percentage of CD3^−^CD19^+^ B cells (3.6 ± 1.1% vs. 12.5 ± 2.1% in controls). Compared with the IMQ group, B-cell frequencies were unchanged in MTX-, CRT-L-, and CRT-M-treated mice but were reduced in the CRT-H group (Figure 4F,I).

### 3.4. CRT Suppresses the Maturation and Activation of BMDCs and Reduces Pro-Inflammatory Cytokine Expression

**Figure 5:**
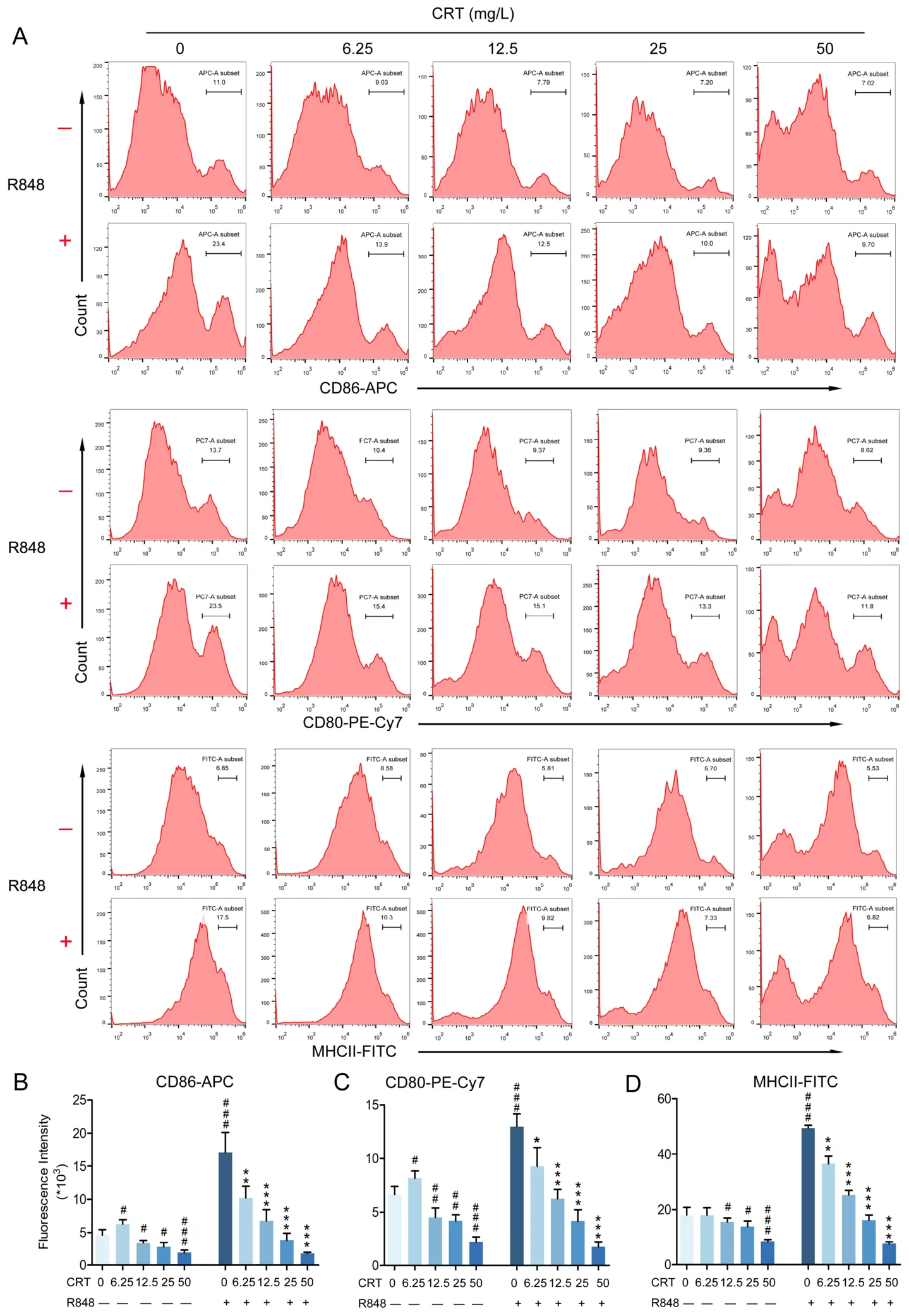
CRT inhibits R848-induced maturation and activation of BMDCs. (**A**) After gating on the CD11c^+^ population, BMDCs were analyzed by flow cytometry to analyze the expression change of CD86/CD80/MHCII on BMDCs that were exposed to R848 and/or different concentrations of CRT. (**B**–**D**) Fluorescence intensity of BMDCs expressing CD86/CD80/MHCII stimulated by R848 and/or different concentration of CRT analyzed statistically. Data are presented as means ± SEM (n = 3). Significance levels: * *p* < 0.05, ** *p* < 0.01 and *** *p* < 0.001 vs. R848 group; # *p* < 0.05, ## *p* < 0.01 and ### *p* < 0.001 vs. vehicle group.

Animal experiments showed that CRT modulates the DC-Th17 axis, and network pharmacology implicated the TLR pathway—a key mechanism in DC-mediated psoriasis. Thus, a BMDC model was used in vitro to determine CRT’s direct action on DC maturation. To exclude cytotoxicity, we first examined the effect of CRT on BMDC viability by using the CCK-8 assay. As shown in Supplementary Figure S2, CRT at concentrations up to 200 µg/mL did not significantly affect cell viability (≥ 90% survival). BMDCs differentiated with IL-4 and GM-CSF were treated with CRT in the presence or absence of R848 (a TLR7/8 agonist) for 24 h. Flow cytometry of CD11c+ cells revealed that R848 markedly increased MHC II, CD80 and CD86 expression, whereas CRT reduced these maturation markers dose-dependently (Figure 5A–D). At 6.25 mg/L, CRT alone slightly upregulated CD80/CD86; at 12.5 mg/L and 25.0 mg/L, CRT moderately decreased marker expression with no significant differences between the two concentrations.

**Figure 6:**
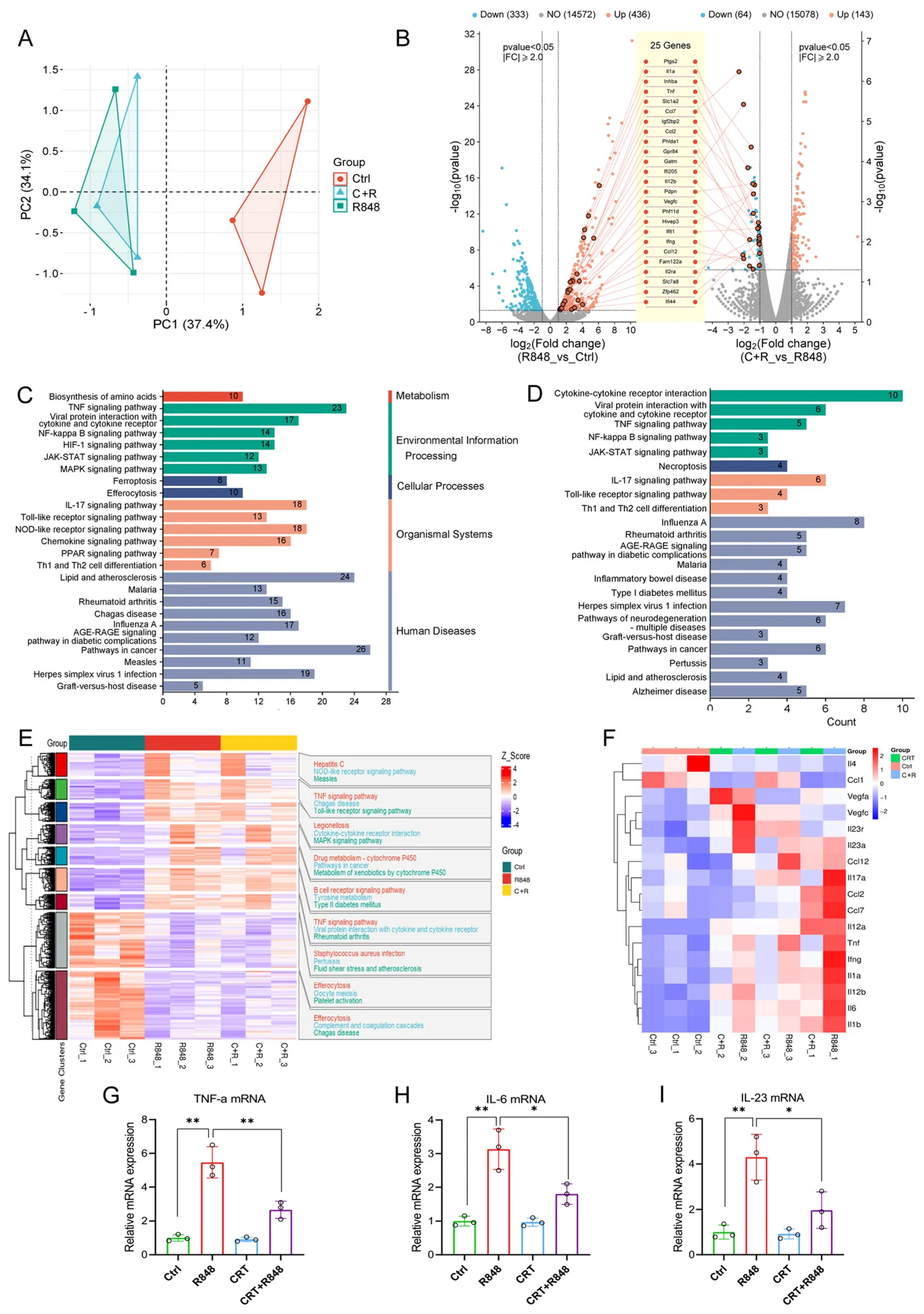
CRT regulates gene expression of R848-stimulated DCs. (**A**) PCA score plot of samples from control, R848-treated, and CRT-treated DCs. (**B**) Double volcano plots displaying DEGs for the comparisons of control vs. R848 and R848 vs. CRT group. (**C**) KEGG enrichment for upregulated DEGs (R848 vs. control). (**D**) KEGG enrichment for DEGs suppressed by CRT. (**E**) Clustering heatmap of all DEGs. (**F**) Heatmap of key inflammatory factors from the samples in DCs. (**G**–**I**) qPCR analysis of (**G**) TNF-a, (**H**) IL-6, and (**I**) IL-23 in DCs. Data are presented as means ± SEM (N = 3). Significance levels: * *p* < 0.05 and ** *p* < 0.01 \ vs. R848 group.

Transcriptome analysis was performed on DCs treated with 12.5 mg/L CRT ± R848 (Figure 6). PCA (Figure 6A) showed distinct separation among the Ctrl, R848, and CRT+R848 groups. R848 induced broad gene upregulation, partially reversed by CRT. Among the three conditions, 36 DEGs were identified; CRT reversed 25 overexpressed and 11 underexpressed genes (Figure 6B). KEGG enrichment of R848-upregulated genes highlighted inflammatory and immune-related pathways, including TNF, NF-κB, HIF-1, JAK-STAT, MAPK, IL-17, TLR and chemokine signaling (Figure 6C). Core pathway analysis of common DEGs further emphasized inflammatory and immune-related pathways, such as cytokine–cytokine receptor interaction, TNF, JAK-STAT, NF-κB, IL-17, and TLR signaling. Cluster heatmaps (Figure 6E,F) demonstrated the CRT regulated members of the interleukin/chemokine family gene families. RT-qPCR validated that CRT (12.5 mg/L) significantly inhibited R848-induced TNF-α, IL-6 and IL-23 mRNA expression (Figure 6G–I). Taken together, network pharmacology and transcriptomics suggest that CRT exerts anti-inflammatory effects via TNF, JAK/STAT3, NF-κB, and MAPK pathways, with key targets including TNFA, STAT3, JUN, RELA, and MAPK.

### 3.5. CRT Inhibited R848-Induced NF-κB Signaling Pathway

**Figure 7:**
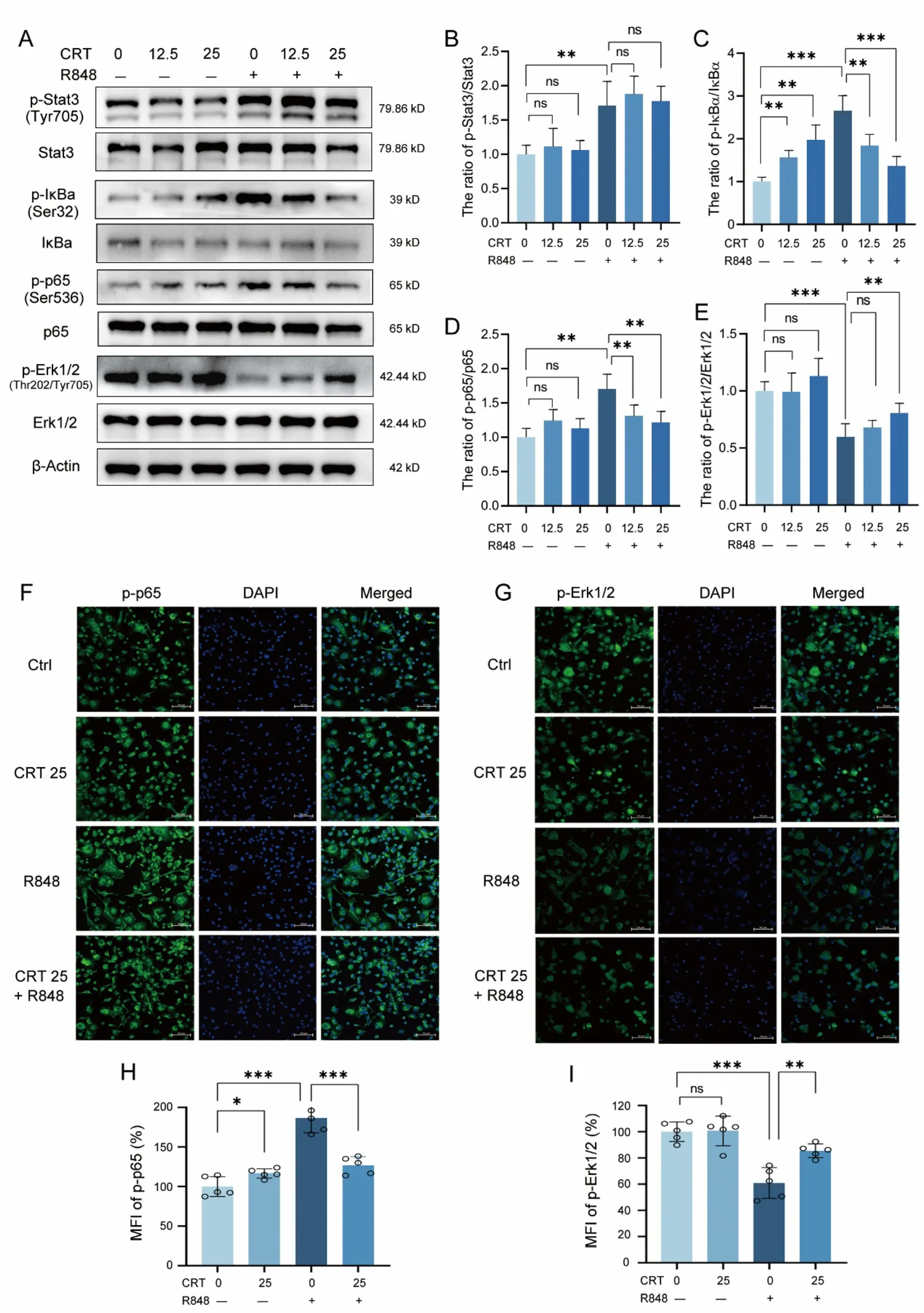
CRT modulates NF-κB signaling in R848-treated DCs. (**A**) Western blot analysis of Stat3, IκBα, p65, Erk1/2, and their phosphorylated forms in R848-induced BMDCs with or without CRT; β-actin served as a loading control. (**B**–**E**) Quantitative analysis of the protein levels of p-Stat3/Stat3, p-IκBα/IκBα, p-p65/p65, and p-Erk1/2/Erk1/2. Western blot data were obtained from three independent biological replicates (n = 3). (**F**,**G**) Immunofluorescence of (**F**) p-p65 and (**G**) p-Erk1/2 expression was detected in BMDCs by exposure to R848 with or without CRT for 12 h (×200; scale bar = 50 μm). (**H**,**I**) Quantitative analysis of mean fluorescence intensity (MFI) of (**H**) p-p65 and (**I**) p-Erk1/2. Data are presented as means ± SEM (n = 5 random fields per group). Significance levels: * *p* < 0.05, ** *p* < 0.01 and *** *p* < 0.001 vs. indicated group.

Based on the transcriptome analysis, we performed Western blotting to examine the effect of CRT on the ERK (MAPK3), STAT3 and NF-κB signaling pathways. The results in Figure 7A–E show that R848 increased the phosphorylation level of Stat3 and NF-κB p65 and IκBα, while it decreased the phosphorylation of Erk1/2 expression in DCs, without altering the total protein level of these molecules. In contrast, CRT treatment attenuated R848-induced phosphorylation of p65 and IκBα (Figure 7C,D), enhanced Erk1/2 (Figure 7E) phosphorylation but did not significantly affect Stat3 phosphorylation (Figure 7B). Consistent with these results, immunofluorescence staining revealed that R848 stimulation induced pronounced nuclear accumulation of p-p65 (Figure 7F,H) and reduced nuclear localization of p-Erk1/2 (Figure 7G,I). Notably, CRT treatment markedly diminished the R848-induced nuclear accumulation of p-p65 (Figure 7F,H) and restored the nuclear distribution of p-Erk1/2 (Figure 7G,I). These observations suggest that CRT inhibited the maturation and activation of BMDCs, at least in part, through suppression of the NF-κB signaling pathway.

### 3.6. Molecular Docking Profiles of CRT-Derived Compounds Targeting RELA

**Figure 8:**
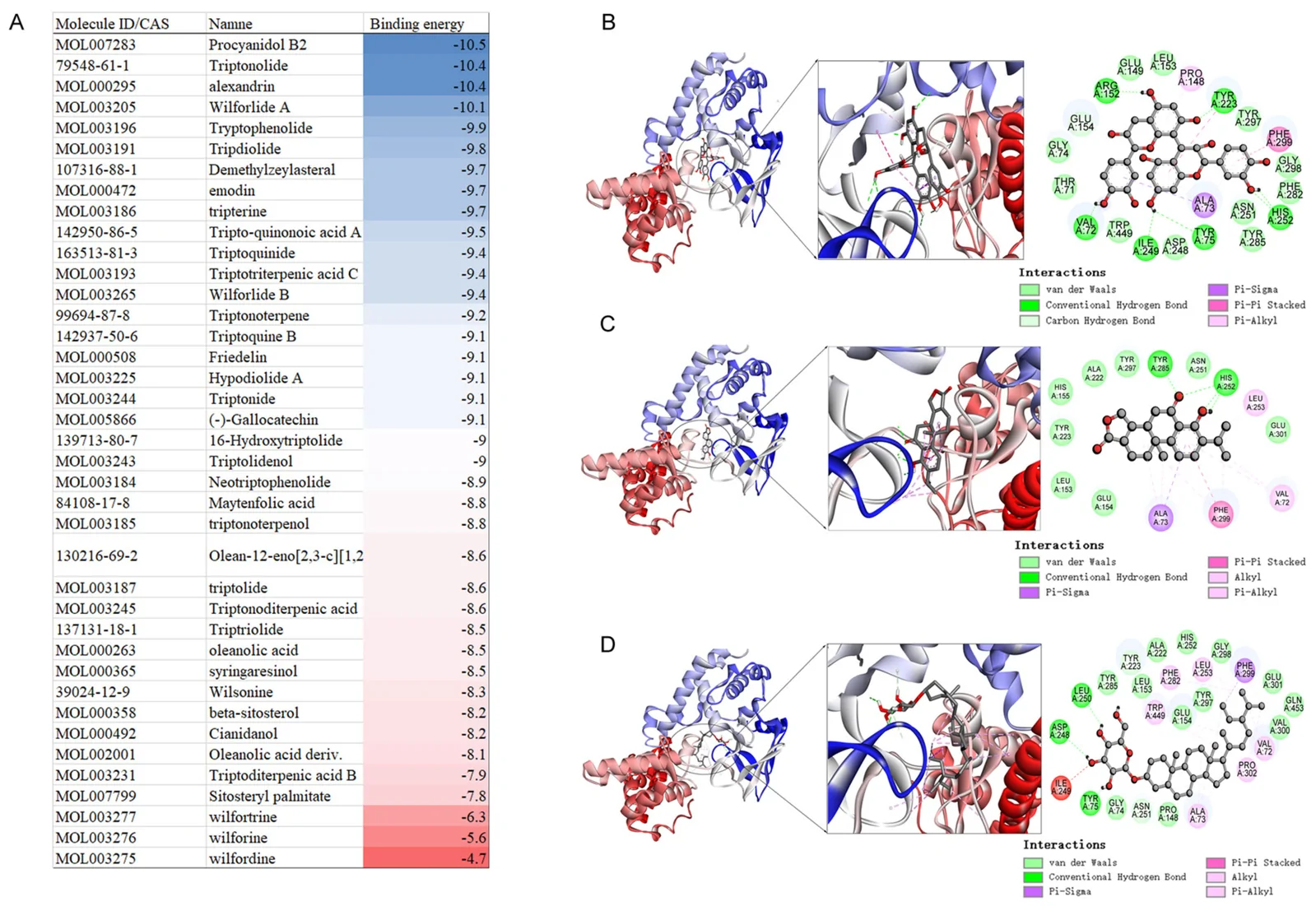
Molecular docking analysis of CRT components with key targets. (**A**) Heatmap of docking scores of RELA (PDB: 3QXY) and 39 ingredients from CRT. (**B**–**D**) Three- and two-dimensional binding modes of RELA and Procyanidol B2 ((**B**); 79548-61-1), Triptonolide ((**C**); MOL007283), and alexandrine ((**D**); MOL000295).

To evaluate the potential binding interactions between the active compounds derived from Tripterygium hypoglaucum (CRT) and the psoriasis-associated transcription factor RELA (p65), molecular docking was performed using the crystal structure of RELA (PDB: 1NFI). A total of 39 bioactive ingredients from CRT were subjected to docking simulations, and their binding energies were calculated (Figure 8A). A lower binding energy indicates a more stable and tighter protein–ligand interaction, with a value below −7 kcal/mol being generally considered indicative of strong binding affinity. Among the 39 compounds, 36 exhibited binding energies lower than −7 kcal/mol, suggesting that the vast majority of CRT components possess considerable binding potential toward RELA. Notably, 22 ingredients displayed binding energies below −9 kcal/mol, and four compounds achieved values lower than −10 kcal/mol, reflecting exceptionally strong interactions. The binding energies of the top six compounds with RELA were as follows: Procyanidol B2, −10.5 kcal/mol (Figure 8B); Triptonolide, −10.4 kcal/mol (Figure 8C); alexandrine, −10.4 kcal/mol (Figure 8D); Wilforlide A, −10.1 kcal/mol; Tryptophenolide, −9.9 kcal/mol; and Tripdiolide, −9.8 kcal/mol. Triptolide, a well-known bioactive component of Tripterygium species, showed a binding energy of −8.6 kcal/mol. Collectively, these results suggest that multiple active constituents of CRT can tightly bind to RELA, with a subset exhibiting particularly outstanding affinity.

## 4. Discussion

In this study, network pharmacology predicted that CRT targets multiple inflammatory pathways, including NF-κB, TLR, and Th17 differentiation, with RELA (p65) as a potential key node. Although several inflammatory pathways were significantly enriched by KEGG analysis, we focused on the Th17 differentiation pathway for subsequent experimental validation. This prioritization was based on the established central role of the Th17/Treg axis in psoriasis pathogenesis and the identification of STAT3, RELA, and JUN as hub targets in our network analysis—all of which are critically involved in Th17 differentiation and function. Experimental validation in an IMQ-induced psoriasis-like mouse model confirmed that CRT-M/H significantly attenuated psoriasiform dermatitis and spleen index (Figure 2), reduced the Th17/Treg ratio, and decreased splenic DCs (Figure 3). Regarding splenomegaly, when corrected for body weight (spleen index = spleen weight/body weight), CRT-M (0.17 g/kg) and CRT-H (0.35 g/kg) significantly reduced the spleen index compared with the IMQ group (*p* < 0.05), whereas the effect of CRT-L did not reach statistical significance. The apparent discrepancy between skin improvement and spleen weight reduction may be partly explained by the marked body weight loss in IMQ-treated mice, which could confound the interpretation of absolute spleen weight. Interestingly, CRT significantly reduced Th17 cells but did not markedly increase Tregs, suggesting that its immunomodulatory effect may be preferentially directed toward suppressing the Th17 axis rather than globally restoring Treg numbers. This observation is consistent with previous reports that certain Tripterygium-derived compounds, including Tripterygium wilfordii polyglycosides and celastrol, preferentially suppress Th17 differentiation [15,16].

These in vivo observations were further supported by in vitro experiments showing that CRT dose-dependently inhibited R848-induced BMDC maturation and suppressed NF-κB phosphorylation (Figure 5, Figure 6 and Figure 7). Transcriptomic profiling suggested that CRT modulated gene expression changes are associated with TNF, JAK-STAT, NF-κB, IL-17, and TLR pathways. RT-qPCR validation confirmed that CRT significantly inhibited R848-induced TNF-α, IL-6, and IL-23A mRNA expression (Figure 6G–I), whereas the broader pathway effects remain to be experimentally validated. Molecular docking suggested that the majority of CRT-derived compounds exhibit strong binding affinity to RELA (Figure 8). Given the central role of NF-κB signaling in bridging innate and adaptive immune responses in psoriasis, we next examined the relevance of this pathway to CRT’s mechanism of action. The nuclear factor kappa-B (NF-κB) signaling pathway plays a pivotal role in the pathogenesis of psoriasis [17]. Its aberrant activation drives a pathological cascade involving dendritic cells (DCs) [18], Th17 cells, and keratinocytes (KCs), making it a highly promising therapeutic target. Current NF-κB-targeting strategies primarily focus on small-molecule inhibitors [19] (e.g., IKK inhibitors [18]). Broad-spectrum NF-κB inhibitors, such as SPC-839 and ML120B, which act by occupying the conserved ATP pocket within the IKK complex, have demonstrated efficacy in ameliorating disease severity in preclinical rheumatoid arthritis models [20]. Notably, the clinical development of SPC-839 by Merck Sorono was subsequently discontinued, possibly due to risks of off-target effects and excessive immunosuppression. Traditional Chinese herbal formulations, with their multi-target properties, may offer a more balanced modulation of NF-κB, potentially facilitating “immune homeostasis restoration.” For instance, triptolide—a bioactive compound derived from Tripterygium wilfordii—exerts selective inhibitory effects on the p65 subunit of NF-κB by blocking its transcriptional activation domain [21,22]. Molecular docking (Figure 8) indicated that procyanidin B and triptonolide exhibited a higher binding affinity than triptolide. Unlike selective IKK inhibitors, which have faced clinical setbacks due to off-target immunosuppression, CRT—as a multi-component herbal formulation—may exert a more balanced modulation of NF-κB signaling. This could potentially reduce the risk of excessive immune suppression while retaining therapeutic efficacy, although the present data suggest that CRT’s immunomodulatory effects are partial rather than fully restorative, consistent with the observed incomplete correction of splenomegaly, Treg levels, and lymphocyte populations.

Although CRT and Tripterygium wilfordii tablets share several bioactive components (e.g., triptolide, tripdiolide, flavonoids, and catechins), CRT is distinguished by the presence of triptophenolide and a lower concentration of triptolide [23], which has been proposed to contribute to its reduced toxicity and enhanced therapeutic efficacy based on the previous literature, although this hypothesis warrants further investigation. Nevertheless, similar to Tripterygium wilfordii, CRT has been reported to exhibit reversible reproductive toxicity [24], including menstrual irregularities, and therefore should be used with caution in patients of reproductive age with fertility concerns.

Currently, research on the mechanism of CRT primarily focuses on its protective effects in diabetic nephropathy. For instance, studies have shown that it can regulate epithelial–mesenchymal transition through the PTEN/PI3K/AKT signaling pathway [25]. Our study suggests that CRT may improve psoriasis by inhibiting NF-κB p65-dependent dendritic-cell maturation, which also provides therapeutic strategies for inflammatory diseases with NF-κB activation.

Several limitations of this study should be acknowledged. First, while our results demonstrate that CRT modulates NF-κB-driven DC maturation in vitro and is associated with Th17/Treg correction in vivo, we did not establish a direct causal link between these two events, nor did we provide in vivo evidence that CRT specifically inhibits NF-κB activity within dendritic cells. Direct causal evidence therefore requires further investigation through DC-specific targeting or co-culture experiments. Second, it is important to note that CRT broadly reduced multiple lymphocyte populations, including CD3^+^, CD4^+^, CD8^+^, and CD19^+^ cells, suggesting that CRT may exert a global immunoregulatory effect on immune-cell homeostasis rather than selectively targeting the Th17 axis alone. While we demonstrated that CRT inhibits NF-κB-driven DC maturation in vitro, the systemic effects observed in vivo raise the possibility that additional immune-cell types or pathways may also contribute to the therapeutic efficacy of CRT in psoriasis. Future studies are needed to dissect the relative contributions of DC-mediated and non-DC-mediated mechanisms. Third, although molecular docking predicted strong binding affinities between multiple CRT-derived compounds and RELA, broader experimental verification of additional bioactive constituents is needed to further strengthen the mechanistic understanding. Fourth, the PASI-like scoring was performed without blinding, which may introduce potential observer bias and should be considered when interpreting the results. Moving forward, we plan to systematically evaluate the therapeutic efficacy of key CRT components in psoriasis models, integrating both DC and Th17 axes to elucidate their combined contributions.

## 5. Conclusions

**Figure 9:**
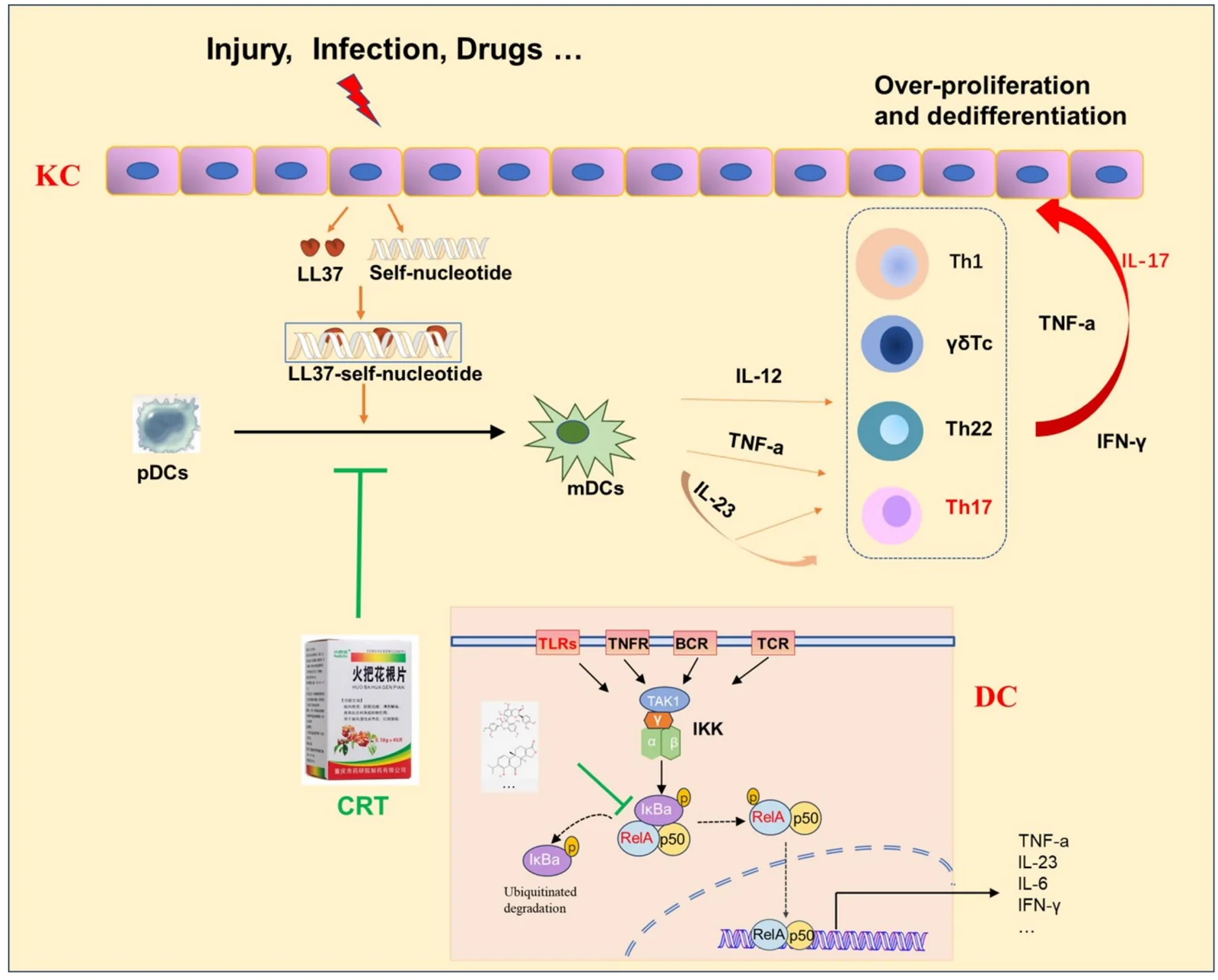
Anti-psoriasis molecular mechanism of CRT.

In summary, CRT exerts anti-psoriatic effects in an IMQ-induced murine model, with concurrent modulation of NF-κB-related dendritic-cell maturation and correction of Th17/Treg imbalance (Figure 9). These findings suggest a potential immunomodulatory mechanism involving both DC-mediated and broader immune-cell effects, though direct causal evidence requires further investigation. The effective dose aligns with its clinical regimen for rheumatoid arthritis, supporting translational feasibility.

## Figures and Tables

**Table 1:** Primer sequences used for RT-qPCR.

Gene	Forward	Reverse
mIL-23a	ACCCACAAGGACTCAAGGAC	CTGCCACTGCTGACTAGAAC
mIL-6	CTGCAAGAGACTTCCATCCAG	AGTGGTATAGACAGGTCTGTTGG
mTNF-α	CCTGTAGCCCACGTCGTAG	GGGAGTAGACAAGGTACAACCC
mβ-actin	GTGACGTTGACATCCGTAAAGA	GCCGGACTCATCGTACTCC

## Data Availability

The data presented in this study are contained within the article and Supplementary Material. Further inquiries can be directed to the corresponding author.

## Supplementary Materials

The following supporting information can be downloaded at: https://www.mdpi.com/article/10.3390/pharmaceutics18091105/s1, Table S1. List of 43 compounds from Colquhounia Root Tablet (CRT) used for network analysis following a review of the literature; a total of 14 compounds were identified by UPLC-Q-TOF-MS, and the others from CNKI. Table S2. List of 179 genes linked with 23 compounds out of a total of 43 compounds. Table S3. List of the interactions between 22 compounds from CRT and psoriasis-related 98 target genes. Figure S1. Identification of the main chemical compounds contained in CRT using UPLC-Q-TOF-MS method. (A,B) CRT. (C) Procyanidol B2. (D) Triptonolide/ Triptoquinide. (E) Triptriolide. (F) Triptonolide and Triptoquinide. (G) Tripdiolide. (H) Triptolide. Figure S2. Effect of CRT on BMDC viability. BMDCs were treated with indicated concentrations of CRT for 24 h, and cell viability was determined by CCK 8 assay. Data are presented as means ± SEM (n = 3 independent experiments). * *p* < 0.05 vs. vehicle group.

## Abbreviations

The following abbreviations are used in this manuscript:

CRT	Colquhounia Root Tablet
THH	Tripterygium hypoglaucum (Lévl.) Hutch
IMQ	Miquimod
MTX	Methotrexate
CRT-L/M/H	Colquhounia Root Tablet at low/middle/high dose
PASI	Psoriasis area and severity index
TCMSP	Traditional Chinese Medicine Systems Pharmacology Database and Analysis Platform
OMIM	Online Mendelian Inheritance in Man
TTD	Therapeutic Target Database
GO	Gene Ontology
KEGG	Kyoto Encyclopedia of Genes and Genomes
STRING	Search Tool for the Retrieval of Interacting Genes/Proteins
PPI	Protein–protein interaction
PDB	Protein Data Bank
UPLC-Q-TOF-MS	Ultra-high-performance liquid chromatography with quadrupole time-of-flight mass spectrometry
EIC	Extracted ion chromatograms
BMDCs	Bone marrow-derived dendritic cells
pDCs	Plasmacytoid dendritic cells
NF-κB	Nuclear factor kappa-B
RELA	NF-κB p65
Stat	Signal transducer and activator of transcription
ERK1/2	MAPK3/MAPK1, mitogen-activated protein kinase 3/
R848	TLR7/TLR8 agonist (Resiquimod)
MFI	Mean fluorescence intensity
