# Supplementary material for: Colquhounia Root Tablet Modulates Psoriatic Immune Responses Involving NF-κB-Driven Dendritic-Cell Maturation and Th17/Treg Imbalance: An Integrative Network Pharmacology and Transcriptomic Study
Source: Pharmaceutics. 2026 Sep 2;18(9):1105. doi: 10.3390/pharmaceutics18091105 (PMC13610900; doi:10.3390/pharmaceutics18091105)
Supplement: Supplementary file 1 [file pharmaceutics-18-01105-s001.zip › pharmaceutics-4410773-supplementary.pdf]

Table S1. List of 43 compounds from Colquhounia Root Tablet (CRT) used for network analysis following a review of the literature; a total of 14 compounds were identified by UPLC-Q-TOF-MS, and the others from CNKI.

Number	Compound	CAS	Source
1	Demethylzeylasteral	107316-88-1	CNKI
2	Triptonide	38647-11-9	CNKI
3	Triptonolide	79548-61-1	CNKI
4	Wilsonine	39024-12-9	CNKI
5	Triptoquinone	163513-81-3	CNKI
6	Triptonoterpene	99694-87-8	CNKI
7	Beta-Sitosterol	83-46-5	CNKI
8	Friedelin	559-74-0	CNKI
9	Emodin	518-82-1	CNKI
10	Neotriptophenolide	81827-74-9	CNKI
11	Daucosterol	474-58-8	CNKI
12	Syringaresinol	1177-14-6	CNKI
13	Triptonoterpenol	110187-23-0	CNKI
14	3-O-Acetyloleanolic acid	4339-72-4	CNKI
15	Triptolidenol	99694-86-7	CNKI
16	16-Hydroxytriptolide	139713-80-7	CNKI
17	Tripdiolide	38647-10-8	CNKI
18	(+) -catechin	154-23-4	CNKI
19	Wilfordine	37239-51-3	CNKI
20	(-)-Gallocatechin	3371-27-5	CNKI
21	Triptonoditerpenic acid	139953-20-1	CNKI
22	Beta-Sitosteryl palmitate	2308-85-2	CNKI
23	Wilforlide A	84104-71-2	CNKI
24	Oleanolic acid	508-02-1	CNKI
25	Wilforlide B	84104-70-1	CNKI
26	Procyanidin B2	29106-49-8	CNKI
27	Wilforgine	37239-47-7	CNKI
28	Integracin B	224186-05-4	CNKI
29	3-oxo-oleanolic acid	130216-69-2	CNKI
30	Triptoquinone A	142950-86-5	UPLC-Q-TOF-MS
31	Wilfortrine	37239-48-8	UPLC-Q-TOF-MS
32	Triptoditerpenic acid	147362-43-4	UPLC-Q-TOF-MS
33	Triptotriterpenic acid A	84108-17-8	UPLC-Q-TOF-MS
34	Triptotriterpenic acid C	123914-32-9	UPLC-Q-TOF-MS
35	Celastrol	34157-83-0	UPLC-Q-TOF-MS
36	Triptoquinone B	142937-50-6	UPLC-Q-TOF-MS
37	Hypodiolide	139122-81-9	UPLC-Q-TOF-MS
38	Triptriolide	137131-18-1	UPLC-Q-TOF-MS
39	Triptolide	38748-32-2	UPLC-Q-TOF-MS
40	Mayteine	104736-05-2	UPLC-Q-TOF-MS

41	Wilfornine A	345954-00-9	UPLC-Q-TOF-MS
42	Wilforine	11088-09-8	UPLC-Q-TOF-MS
43	Triptophenolide	74285-86-2	UPLC-Q-TOF-MS

Table S2. List of 179 genes linked with 23 compounds out of a total of 43 compounds.

Compound	Gene
Triptoditerpenic acid B	PTGS1
Triptoditerpenic acid B	CHRM3
Triptoditerpenic acid B	Kcnh2
Triptoditerpenic acid B	CHRM1
Triptoditerpenic acid B	SCN5A
Triptoditerpenic acid B	F10
Triptoditerpenic acid B	CHRM5
Triptoditerpenic acid B	PTGS2
Triptoditerpenic acid B	CA2
Triptoditerpenic acid B	CHRM4
Triptoditerpenic acid B	RXRA
Triptoditerpenic acid B	OPRD1
Triptoditerpenic acid B	PDE3A
Triptoditerpenic acid B	ADRA1A
Triptoditerpenic acid B	PGR
Triptoditerpenic acid B	CHRM2
Triptoditerpenic acid B	ADRA1B
Triptoditerpenic acid B	ADRB2
Triptoditerpenic acid B	ADRA1D
Triptoditerpenic acid B	TOP2
Triptoditerpenic acid B	OPRM1
Triptoditerpenic acid B	NR3C1
Triptoditerpenic acid B	HSP90AA1，HSP90AB1
Triptoditerpenic acid B	RXRB
Triptoditerpenic acid B	NCOA2
Triptoditerpenic acid B	NCOA1
Celastrol	PTGS2
Celastrol	BIRC5
Celastrol	BIRC2
Celastrol	CFLAR
Celastrol	AKR1B1
Celastrol	CDC37
Celastrol	BCL2
Celastrol	BAX
Celastrol	MMP2

Celastrol	TIMP1
Celastrol	NR3C1
Celastrol	VEGFA
Celastrol	CCND1
Celastrol	BCL2L1
Celastrol	FLT1
Celastrol	KDR
Celastrol	MMP9
Celastrol	JUN
Celastrol	TIMP2
Celastrol	MMP1
Celastrol	MYC
Celastrol	FN1
Celastrol	COL7A1
Celastrol	TGFB1
Celastrol	COL4A4
Celastrol	TLR9
Celastrol	PTPN11
Hypodiolide	NR3C2
Hypodiolide	NR3C1
Triptolide	RELA
Triptolide	STAT3
Triptolide	VEGFA
Triptolide	BCL2
Triptolide	FOS
Triptolide	CDKN1A
Triptolide	PLAU
Triptolide	TNF
Triptolide	JUN
Triptolide	CASP3
Triptolide	TP53
Triptolide	MAPK8
Triptolide	PTGS2
Triptolide	STAT1
Triptolide	CXCL8
Triptolide	MCL1
Triptolide	TGFB1
Triptolide	IL2

Triptolide	IFNG
Triptolide	IL4
Triptolide	XIAP
Triptolide	DEFB4A
Triptolide	CD80
Triptolide	CD86
Triptolide	CXCR4
Triptolide	BIRC3
Triptolide	CD274
Triptolide	IL23A
Triptolide	CCR7
Triptolide	CD1A
Triptolide	CD40
Triptolide	CD14
Triptolide	C3
Triptolide	VTCN1
Triptophenolide	DRD1
Triptophenolide	CHRM3
Triptophenolide	Kcnh2
Triptophenolide	CHRM1
Triptophenolide	SCN5A
Triptophenolide	CHRM5
Triptophenolide	PTGS2
Triptophenolide	CA2
Triptophenolide	RXRA
Triptophenolide	OPRD1
Triptophenolide	PDE3A
Triptophenolide	ADRA1A
Triptophenolide	PGR
Triptophenolide	CHRM2
Triptophenolide	ADRA1B
Triptophenolide	ADRB2
Triptophenolide	ADRA1D
Triptophenolide	TOP2
Triptophenolide	OPRM1
Triptophenolide	HSP90AA1 and HSP90AB1
Triptophenolide	CHRNA7
Triptophenolide	NCOA2

Triptophenolide	NCOA1
Triptonolide	CHRM3
Triptonolide	CHRM1
Triptonolide	SCN5A
Triptonolide	F10
Triptonolide	CHRM5
Triptonolide	PTGS2
Triptonolide	CA2
Triptonolide	OPRD1
Triptonolide	ADRA1A
Triptonolide	PGR
Triptonolide	CHRM2
Triptonolide	ADRB2
Triptonolide	TOP2
Triptonolide	OPRM1
Triptonolide	CHRNA7
Triptonolide	NCOA2
Triptonolide	NCOA1
Triptonolide	CALM1
Wilsonine	DRD1
Wilsonine	CHRM3
Wilsonine	CHRM1
Wilsonine	AR
Wilsonine	DRD5
Wilsonine	SCN5A
Wilsonine	CHRM5
Wilsonine	HTR3A
Wilsonine	CHRM4
Wilsonine	OPRD1
Wilsonine	ACHE
Wilsonine	HTR2A
Wilsonine	ADRA1A
Wilsonine	HTR2C
Wilsonine	CHRM2
Wilsonine	ADRA1B
Wilsonine	ADRB2
Wilsonine	ADRA1D
Wilsonine	CHRNA2

Wilsonine	SLC6A4
Wilsonine	DRD2
Wilsonine	OPRM1
Wilsonine	GABRA1
Triptonoterpene	PTGS1
Triptonoterpene	CHRM3
Triptonoterpene	CHRM1
Triptonoterpene	SCN5A
Triptonoterpene	PTGS2
Triptonoterpene	CA2
Triptonoterpene	RXRA
Triptonoterpene	ACHE
Triptonoterpene	ADRA1A
Triptonoterpene	PGR
Triptonoterpene	CHRM2
Triptonoterpene	ADRA1B
Triptonoterpene	ADRB2
Triptonoterpene	ADRA1D
Triptonoterpene	OPRM1
Triptonoterpene	NR3C1
Triptonoterpene	NCOA2
Triptonoterpene	NCOA1
Beta-Sitosterol	PGR
Beta-Sitosterol	NCOA2
Beta-Sitosterol	PTGS1
Beta-Sitosterol	PTGS2
Beta-Sitosterol	HSP90AA1 and HSP90AB1
Beta-Sitosterol	PIK3CG
Beta-Sitosterol	Kcnh2
Beta-Sitosterol	PRKACA
Beta-Sitosterol	DRD1
Beta-Sitosterol	CHRM3
Beta-Sitosterol	CHRM1
Beta-Sitosterol	SCN5A
Beta-Sitosterol	GABRA2
Beta-Sitosterol	CHRM4
Beta-Sitosterol	PDE3A
Beta-Sitosterol	HTR2A

Beta-Sitosterol	GABRA5
Beta-Sitosterol	ADRA1A
Beta-Sitosterol	GABRA3
Beta-Sitosterol	CHRM2
Beta-Sitosterol	ADRA1B
Beta-Sitosterol	ADRB2
Beta-Sitosterol	CHRNA2
Beta-Sitosterol	SLC6A4
Beta-Sitosterol	OPRM1
Beta-Sitosterol	GABRA1
Beta-Sitosterol	CHRNA7
Beta-Sitosterol	Cytochrome P450-cam
Beta-Sitosterol	BCL2
Beta-Sitosterol	BAX
Beta-Sitosterol	CASP9
Beta-Sitosterol	JUN
Beta-Sitosterol	CASP3
Beta-Sitosterol	CASP8
Beta-Sitosterol	PRKCA
Beta-Sitosterol	TGFB1
Beta-Sitosterol	PON1
Beta-Sitosterol	MAP2
Beta-Sitosterol	HMGCR
Beta-Sitosterol	CYP51A1
Beta-Sitosterol	AR
Beta-Sitosterol	NPC1L1
Beta-Sitosterol	NR1H3
Beta-Sitosterol	CYP17A1
Beta-Sitosterol	RORC
Friedelin	CYP19A1
Friedelin	CA2
Friedelin	CA1
Friedelin	CA4
Emodin	PTGS1
Emodin	PTGS2
Emodin	F7
Emodin	HSP90AA1 and HSP90AB1
Emodin	PIK3CG

Emodin	PRKACA
Emodin	IGHG1
Emodin	F10
Emodin	KDR
Emodin	TOP2
Emodin	NCOA2
Emodin	NCOA1
Emodin	CALM1
Emodin	CDKN1A
Emodin	FLT1
Emodin	MMP9
Emodin	EGF
Emodin	TNF
Emodin	CASP3
Emodin	TP53
Emodin	PRKCE
Emodin	MMP1
Emodin	PPARG
Emodin	MYC
Emodin	CYP1A1
Emodin	IL1B
Emodin	PRKCD
Emodin	CSF2
Emodin	TGFB1
Emodin	ACTA2
Emodin	MAOB
Emodin	BTK
Emodin	SLC2A4
Emodin	FLT4
Emodin	SLC2A1
Emodin	ESR1
Emodin	PIM1
Emodin	ESR2
Emodin	CSNK2A1
Emodin	PTP4A3
Emodin	VEGFA
Emodin	CYP1B1
Emodin	ERBB2

Emodin	LCK
Emodin	MCL1
Emodin	CXCR4
Neotriptophenolide	PTGS1
Neotriptophenolide	CHRM3
Neotriptophenolide	Kcnh2
Neotriptophenolide	CHRM1
Neotriptophenolide	SCN5A
Neotriptophenolide	F10
Neotriptophenolide	CHRM5
Neotriptophenolide	PTGS2
Neotriptophenolide	CA2
Neotriptophenolide	CHRM4
Neotriptophenolide	OPRD1
Neotriptophenolide	PGR
Neotriptophenolide	CHRM2
Neotriptophenolide	ADRA1B
Neotriptophenolide	ADRB2
Neotriptophenolide	TOP2
Neotriptophenolide	OPRM1
Neotriptophenolide	HSP90AA1 and HSP90AB1
Neotriptophenolide	NCOA2
Neotriptophenolide	NCOA1
Neotriptophenolide	KCNMA1
Daucosterol	IL2
Syringaresinol	F10
Syringaresinol	PTGS2
Syringaresinol	TOP2
Syringaresinol	NCOA2
Syringaresinol	CALM1
Syringaresinol	Kcnh2
Syringaresinol	F7
Syringaresinol	KCNMA1
Syringaresinol	HSP90AA1 and HSP90AB1
Triptonoterpenol	CHRM3
Triptonoterpenol	CHRM1
Triptonoterpenol	PTGS2
Triptonoterpenol	CA2

Triptonoterpenol	OPRD1
Triptonoterpenol	ADRA1A
Triptonoterpenol	ADRA1B
Triptonoterpenol	ADRA1D
Triptonoterpenol	TOP2
Triptonoterpenol	OPRM1
Triptonoterpenol	NR3C1
Triptonoterpenol	NCOA2
Triptonoterpenol	NCOA1
Triptonoterpenol	SCN5A
Triptonoterpenol	CHRM2
Triptonoterpenol	ADRB2
Triptonoterpenol	HSP90AA1 and HSP90AB1
3-O-Acetyloleanolic acid	PTPN1
Tripdiolide	TRIAP1
(+)-catechin	PTGS1
(+)-catechin	ESR1
(+)-catechin	PTGS2
(+)-catechin	HSP90AA1 and HSP90AB1
(+)-catechin	LACTB
(+)-catechin	PRKACA
(+)-catechin	NCOA2
(+)-catechin	CALM1
(+)-catechin	RXRA
(+)-catechin	CAT
(+)-catechin	HAS2
Wilfordine	HMOX1
Wilfordine	DNMT1
Wilfordine	PTGS2
Wilfordine	SLC47A1
Wilfordine	APOB
Wilfordine	PON1
Wilfordine	KIAA1149
Wilfordine	SLC22A11
Wilfordine	BACE1
Wilfordine	CSF2
(-)-Gallocatechin	PTGS1
(-)-Gallocatechin	ESR1

(-)-Gallocatechin	PTGS2
(-)-Gallocatechin	TOP2
(-)-Gallocatechin	HSP90AA1 and HSP90AB1
(-)-Gallocatechin	LACTB
(-)-Gallocatechin	NCOA2
(-)-Gallocatechin	NR3C1
Triptonoditerpenic acid	CHRM3
Triptonoditerpenic acid	Kcnh2
Triptonoditerpenic acid	CHRM1
Triptonoditerpenic acid	SCN5A
Triptonoditerpenic acid	PTGS2
Triptonoditerpenic acid	CA2
Triptonoditerpenic acid	OPRD1
Triptonoditerpenic acid	ADRA1B
Triptonoditerpenic acid	ADRB2
Triptonoditerpenic acid	TOP2
Triptonoditerpenic acid	NCOA2
Triptonoditerpenic acid	NCOA1
Oleanolic acid	PTGS1
Oleanolic acid	PTGS2
Oleanolic acid	PTPN1
Oleanolic acid	POLB
Oleanolic acid	AKR1B10
Oleanolic acid	RORC
Oleanolic acid	PTPRF
Oleanolic acid	PTPN2
Oleanolic acid	HSD11B1
Oleanolic acid	ACP1
Oleanolic acid	PDE4D
Oleanolic acid	CDC25B
Oleanolic acid	PLA2G1B
Oleanolic acid	CD81
Oleanolic acid	UGT2B10
Oleanolic acid	UGT2B11
Oleanolic acid	NFE2L2
Oleanolic acid	NQO1
Oleanolic acid	PPARA
Oleanolic acid	PTGIR

Oleanolic acid	NAMPT
Oleanolic acid	CASP3
Oleanolic acid	TOP1
Oleanolic acid	TOP2A
Procyanidin B2	MMP14
Procyanidin B2	MAP2K1
Procyanidin B2	PTGS2
Procyanidin B2	PRKCA
Procyanidin B2	PRKCB
Procyanidin B2	MAP2K4
Procyanidin B2	MMP2
Procyanidin B2	PGF
Procyanidin B2	VEGFA
Procyanidin B2	NLRP3
Procyanidin B2	MAPK1
Procyanidin B2	AKT1
Procyanidin B2	MAPK3
Procyanidin B2	TOP2A
Procyanidin B2	CASP9

Table S3. List of the interactions between 22 compounds from CRT and psoriasis-related 98 target genes.

Compound	Target
(-)-Gallocatechin	PTGS1
(-)-Gallocatechin	ESR1
(-)-Gallocatechin	PTGS2
(-)-Gallocatechin	NR3C1
(-)-Gallocatechin	HSP90AA1
(-)-Gallocatechin	HSP90AB1
(+)-catechin	PTGS1
(+)-catechin	ESR1
(+)-catechin	PTGS2
(+)-catechin	RXRA
(+)-catechin	CAT
(+)-catechin	HSP90AA1
(+)-catechin	HSP90AB1
3-O-Acetyloleanolic acid	PTPN1
Beta-Sitosterol	PGR
Beta-Sitosterol	PTGS1
Beta-Sitosterol	PTGS2
Beta-Sitosterol	PIK3CG
Beta-Sitosterol	PDE3A
Beta-Sitosterol	HTR2A
Beta-Sitosterol	ADRA1B
Beta-Sitosterol	SLC6A4
Beta-Sitosterol	OPRM1
Beta-Sitosterol	CHRNA7
Beta-Sitosterol	BCL2
Beta-Sitosterol	BAX
Beta-Sitosterol	JUN
Beta-Sitosterol	CASP3
Beta-Sitosterol	PRKCA
Beta-Sitosterol	TGFB1
Beta-Sitosterol	PON1
Beta-Sitosterol	NR1H3
Beta-Sitosterol	RORC
Beta-Sitosterol	HSP90AA1
Beta-Sitosterol	HSP90AB1
Celastrol	PTGS2
Celastrol	BIRC5
Celastrol	BIRC2
Celastrol	CFLAR
Celastrol	BCL2
Celastrol	BAX
Celastrol	MMP2
Celastrol	TIMP1
Celastrol	NR3C1
Celastrol	VEGFA
Celastrol	CCND1
Celastrol	BCL2

Celastrol	BCL2L1
Celastrol	BAX
Celastrol	FLT1
Celastrol	KDR
Celastrol	MMP9
Celastrol	JUN
Celastrol	TIMP2
Celastrol	MMP1
Celastrol	MYC
Celastrol	FN1
Celastrol	TGFB1
Celastrol	TLR9
Celastrol	PTPN11
Daucosterol	IL2
Emodin	PTGS1
Emodin	PTGS2
Emodin	PIK3CG
Emodin	KDR
Emodin	CDKN1A
Emodin	FLT1
Emodin	MMP9
Emodin	EGF
Emodin	TNF
Emodin	CASP3
Emodin	TP53
Emodin	MMP1
Emodin	PPARG
Emodin	MYC
Emodin	CYP1A1
Emodin	IL1B
Emodin	CSF2
Emodin	TGFB1
Emodin	ACTA2
Emodin	FLT4
Emodin	SLC2A1
Emodin	ESR1
Emodin	ESR2
Emodin	VEGFA
Emodin	CYP1B1
Emodin	TP53
Emodin	TNF
Emodin	CXCR4
Emodin	CASP3
Emodin	HSP90AA1
Emodin	HSP90AB1
Friedelin	CA2
Hypodiolide	NR3C1
Neotriptophenolide	PTGS1
Neotriptophenolide	PTGS2
Neotriptophenolide	CA2

Neotriptophenolide	PGR
Neotriptophenolide	ADRA1B
Neotriptophenolide	OPRM1
Neotriptophenolide	HSP90AA1
Neotriptophenolide	HSP90AB1
Oleanolic acid	PTGS1
Oleanolic acid	PTGS2
Oleanolic acid	PTPN1
Oleanolic acid	PTPN1
Oleanolic acid	AKR1B10
Oleanolic acid	RORC
Oleanolic acid	PTPN2
Oleanolic acid	ACP1
Oleanolic acid	PDE4D
Oleanolic acid	PLA2G1B
Oleanolic acid	NFE2L2
Oleanolic acid	NQO1
Oleanolic acid	PPARA
Oleanolic acid	NAMPT
Oleanolic acid	CASP3
Procyanidin B2	VEGFA
Procyanidin B2	NLRP3
Procyanidin B2	MAPK1
Procyanidin B2	AKT1
Procyanidin B2	MAPK3
Procyanidin B2	PTGS2
Procyanidin B2	PRKCA
Procyanidin B2	PRKCB
Procyanidin B2	MMP2
Procyanidin B2	PGF
Syringaresinol	PTGS2
Syringaresinol	HSP90AA1
Syringaresinol	HSP90AB1
Triptoditerpenic acid B	PTGS1
Triptoditerpenic acid B	PTGS2
Triptoditerpenic acid B	CA2
Triptoditerpenic acid B	RXRA
Triptoditerpenic acid B	PDE3A
Triptoditerpenic acid B	PGR
Triptoditerpenic acid B	ADRA1B
Triptoditerpenic acid B	OPRM1
Triptoditerpenic acid B	NR3C1
Triptoditerpenic acid B	RXRB
Triptoditerpenic acid B	HSP90AA1
Triptoditerpenic acid B	HSP90AB1
Triptolide	RELA
Triptolide	STAT3
Triptolide	VEGFA
Triptolide	BCL2
Triptolide	FOS

Triptolide	CDKN1A
Triptolide	PLAU
Triptolide	TNF
Triptolide	JUN
Triptolide	CASP3
Triptolide	TP53
Triptolide	MAPK8
Triptolide	PTGS2
Triptolide	STAT1
Triptolide	CXCL8
Triptolide	TGFB1
Triptolide	IL2
Triptolide	IFNG
Triptolide	IL4
Triptolide	DEFB4A
Triptolide	CD80
Triptolide	CD86
Triptolide	CXCR4
Triptolide	CD274
Triptolide	IL23A
Triptolide	CCR7
Triptolide	CD1A
Triptolide	CD40
Triptolide	CD14
Triptolide	C3
Triptonoditerpenic acid	PTGS2
Triptonoditerpenic acid	CA2
Triptonoditerpenic acid	ADRA1B
Triptonolide	PTGS2
Triptonolide	CA2
Triptonolide	PGR
Triptonolide	OPRM1
Triptonolide	CHRNA7
Triptonoterpene	PTGS1
Triptonoterpene	PTGS2
Triptonoterpene	CA2
Triptonoterpene	RXRA
Triptonoterpene	PGR
Triptonoterpene	ADRA1B
Triptonoterpene	OPRM1
Triptonoterpene	NR3C1
Triptonoterpenol	PTGS2
Triptonoterpenol	CA2
Triptonoterpenol	ADRA1B
Triptonoterpenol	OPRM1
Triptonoterpenol	NR3C1
Triptonoterpenol	HSP90AA1
Triptonoterpenol	HSP90AB1
Triptophenolide	PTGS2
Triptophenolide	CA2

Triptophenolide	RXRA
Triptophenolide	PDE3A
Triptophenolide	PGR
Triptophenolide	ADRA1B
Triptophenolide	OPRM1
Triptophenolide	CHRNA7
Triptophenolide	HSP90AA1
Triptophenolide	HSP90AB1
Wilfordine	HMOX1
Wilfordine	PTGS2
Wilfordine	APOB
Wilfordine	PON1
Wilfordine	CSF2
Wilsonine	HTR3A
Wilsonine	HTR2A
Wilsonine	HTR2C
Wilsonine	ADRA1B
Wilsonine	SLC6A4
Wilsonine	OPRM1

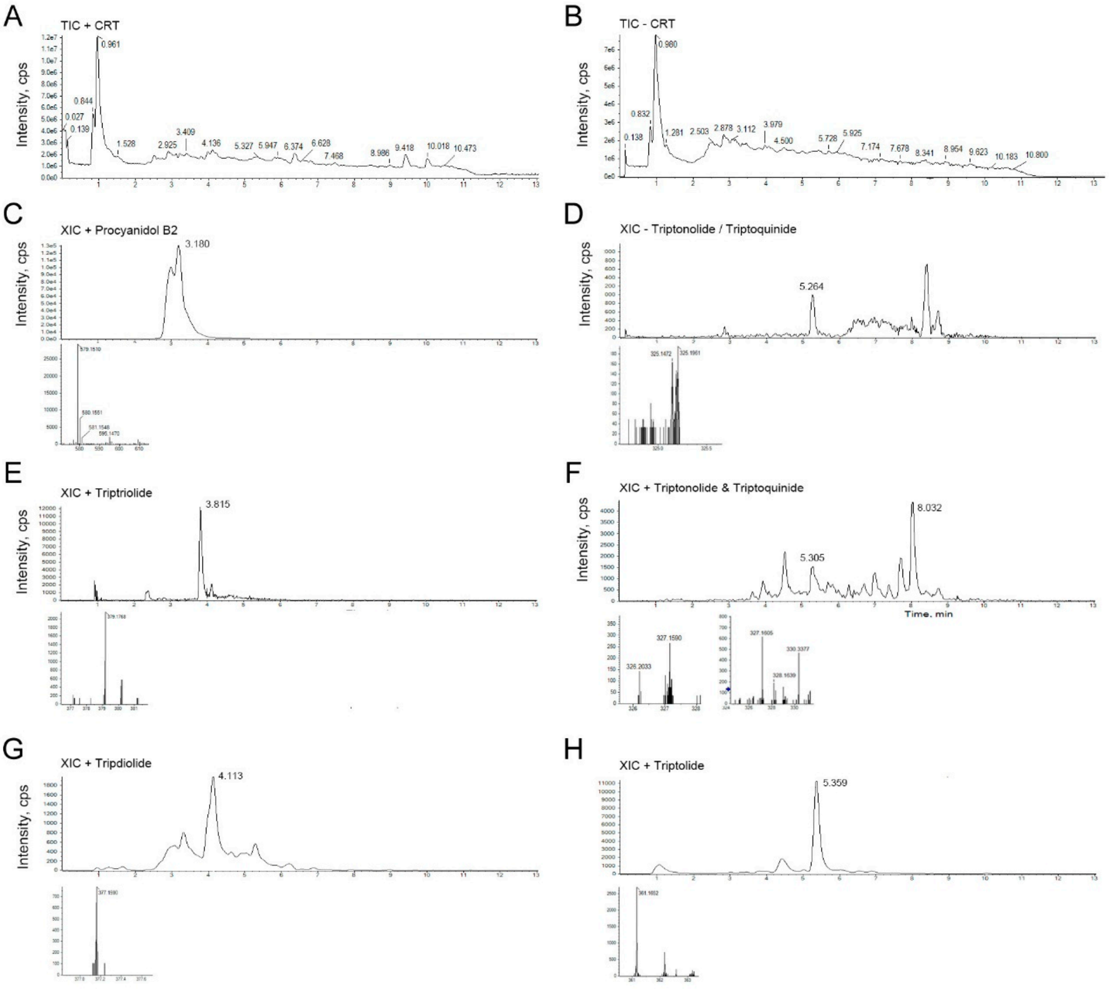

Supplementary Figure S1. Identification of the main chemical compounds contained in CRT using UPLC-Q-TOF-MS method. (A) (B) CRT. (C) Procyanidol B2. (D) Triptonolide/ Triptoquinide. (E) Triptriolide. (F) Triptonolide and Triptoquinide. (G) Tripdiolide. (H) Triptolide.

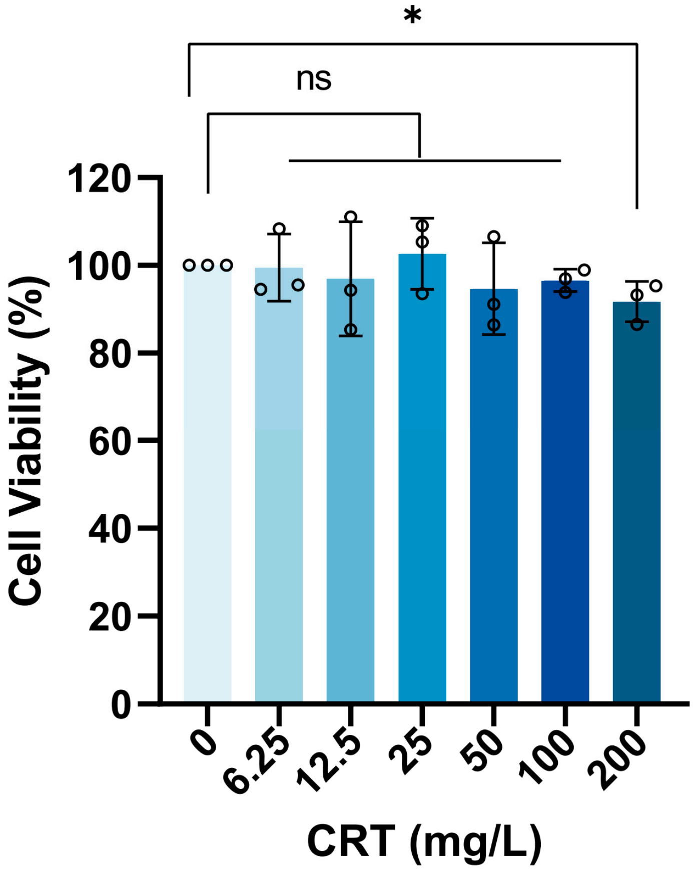

Supplementary Figure S2. Effect of CRT on BMDC viability. BMDCs were treated with indicated concentrations of CRT for 24 h, and cell viability was determined by CCK-8 assay. Data are presented as means ± SEM (n = 3 independent experiments). * P < 0.05 vs. vehicle group.
